# Supplementary figures and images for: Quantifying prevalence and risk factors of HIV multiple infection in Uganda from population-based deep-sequence data
Source: PLoS Pathog. 2025 Apr 22;21(4):e1013065. doi: 10.1371/journal.ppat.1013065 (PMC12055032; doi:10.1371/journal.ppat.1013065)

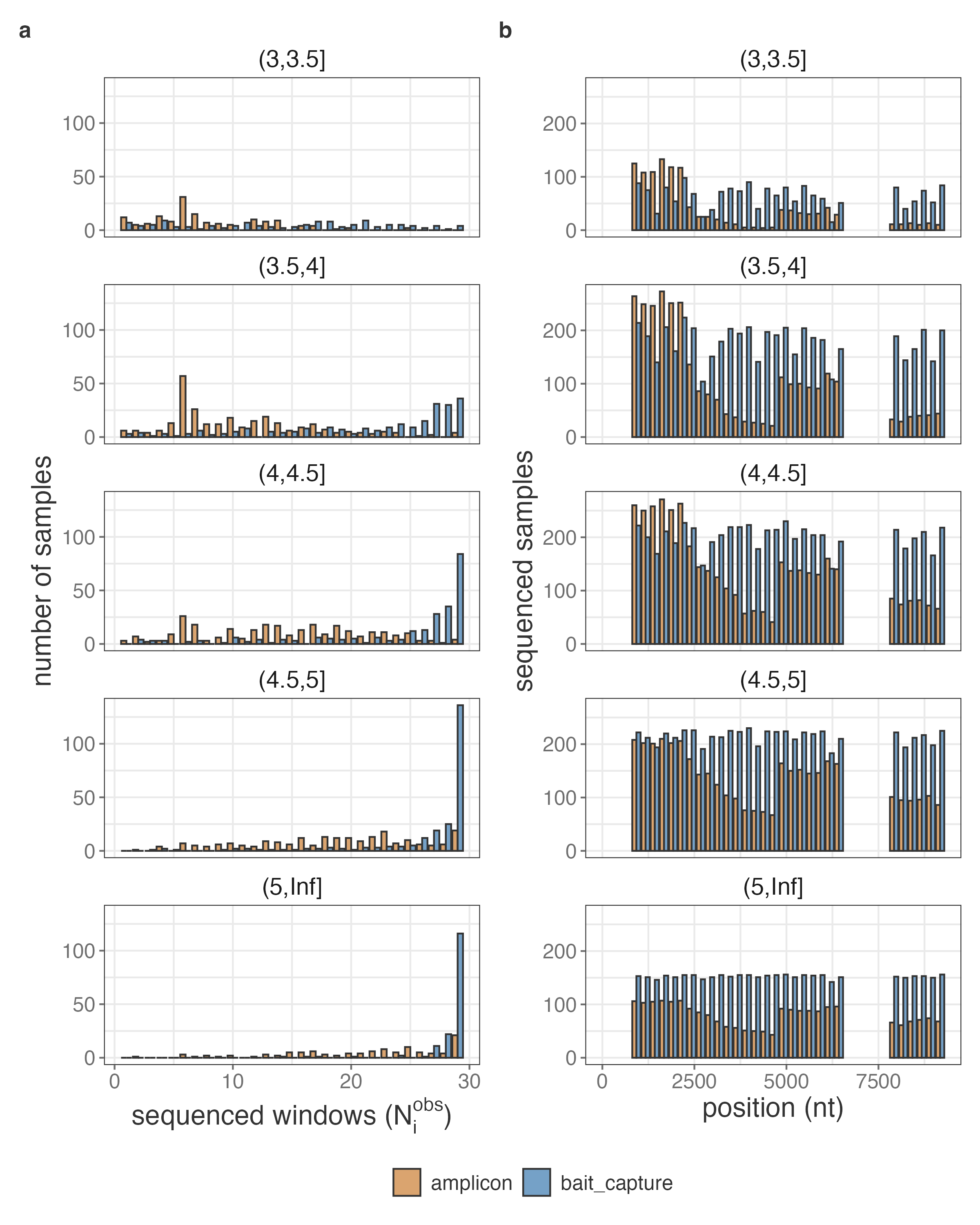

Supplement: S1 Fig — (A) Distribution of Niobs values for all samples. (B) Number of samples with coverage in each of the 29 genome window. (TIF) [file ppat.1013065.s001.tif]

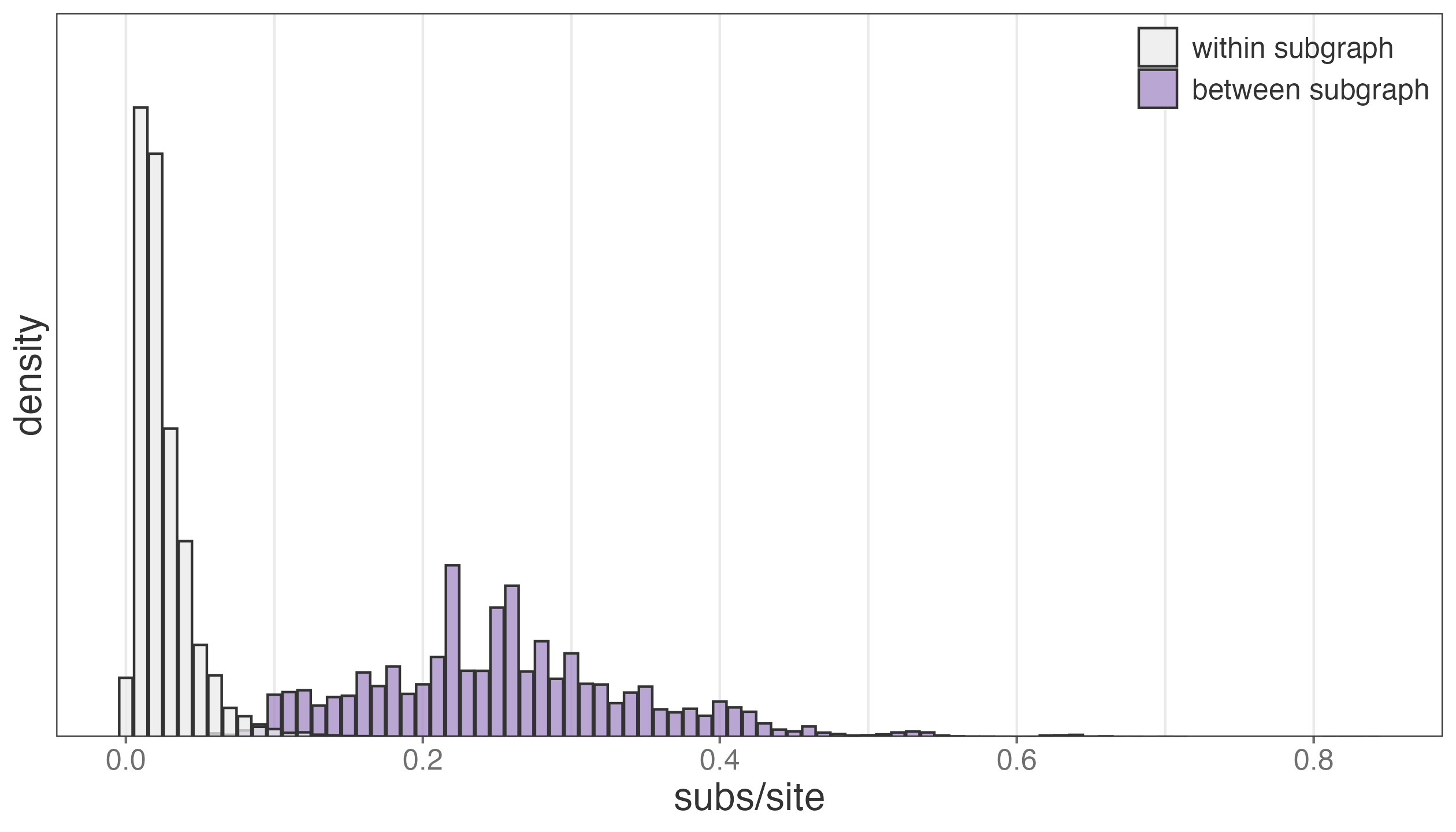

Supplement: S2 Fig — Bins are shaded based on whether tips were assigned to the same subgraph (grey) or different subgraphs (purple), in the case where multiple subgraphs were observed. (TIF) [file ppat.1013065.s002.tif]

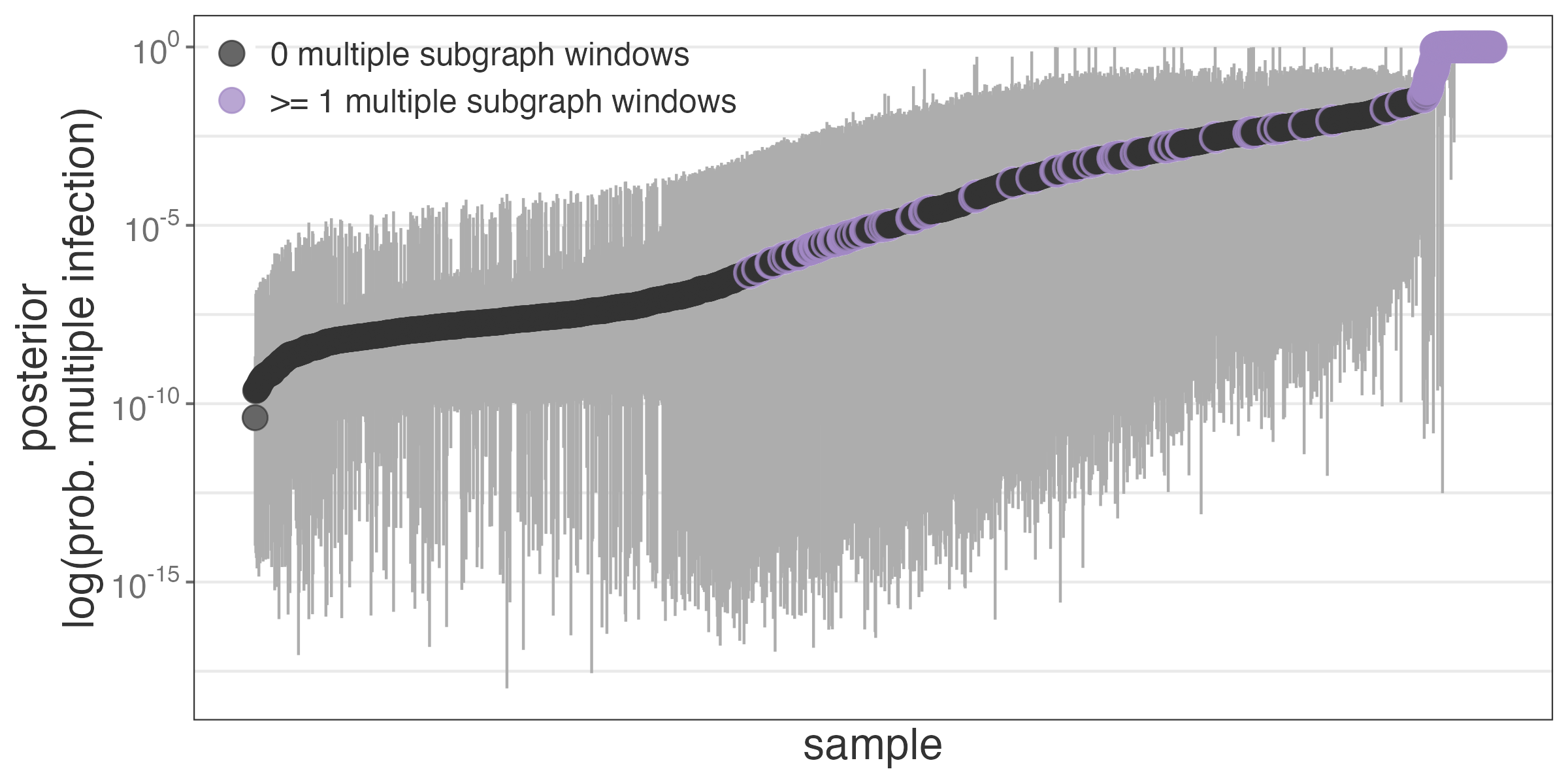

Supplement: S6 Fig — Estimated posterior log 10 probability of multiple infection for each participant. Confidence bounds represent the 95% highest posterior density. Participants with at least one multiple subgraph window are shown in purple. (TIF) [file ppat.1013065.s006.tif]

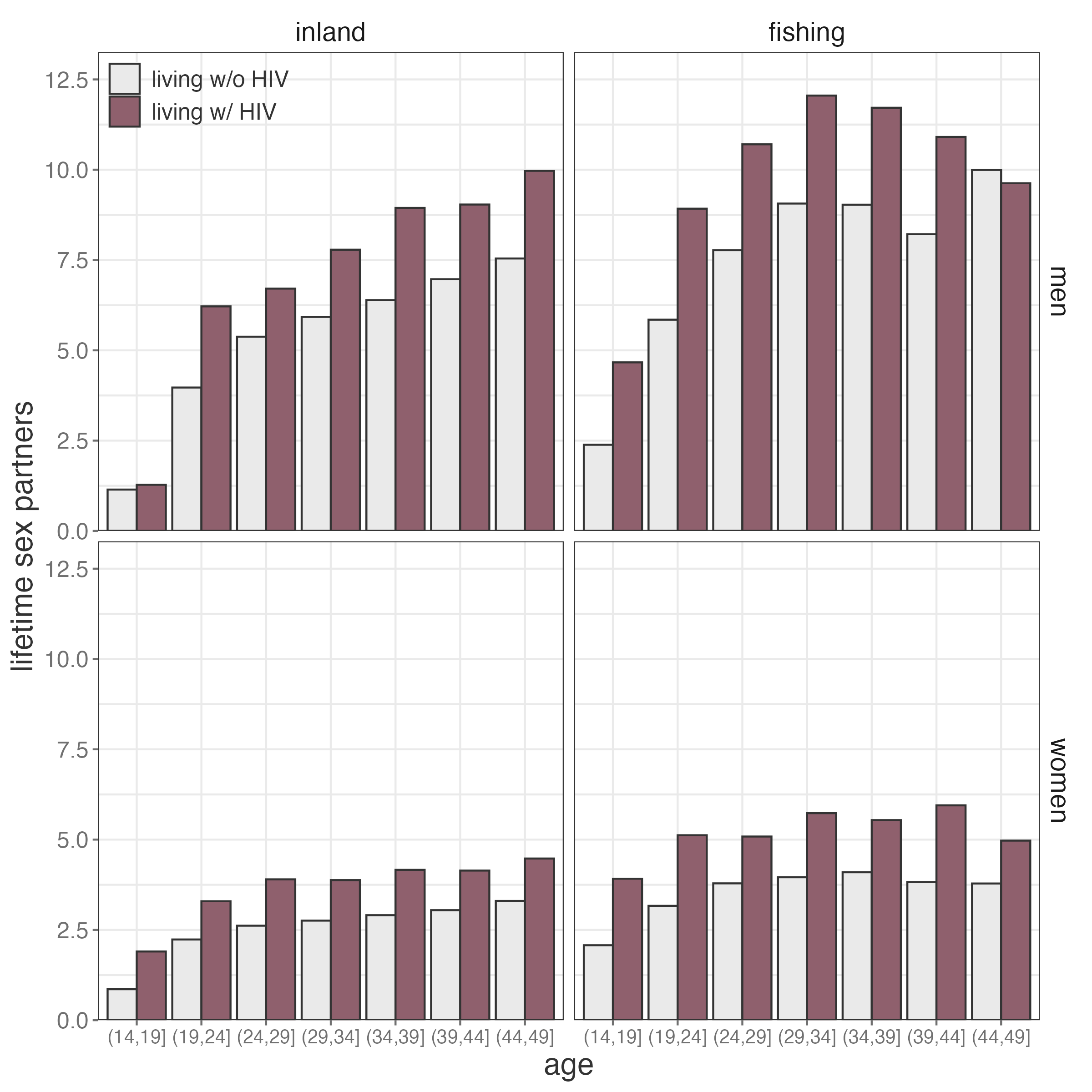

Supplement: S7 Fig — Excludes participant visits in which respondents provided a categorical response (N = 5,436 (10.67%)). (TIF) [file ppat.1013065.s007.tif]

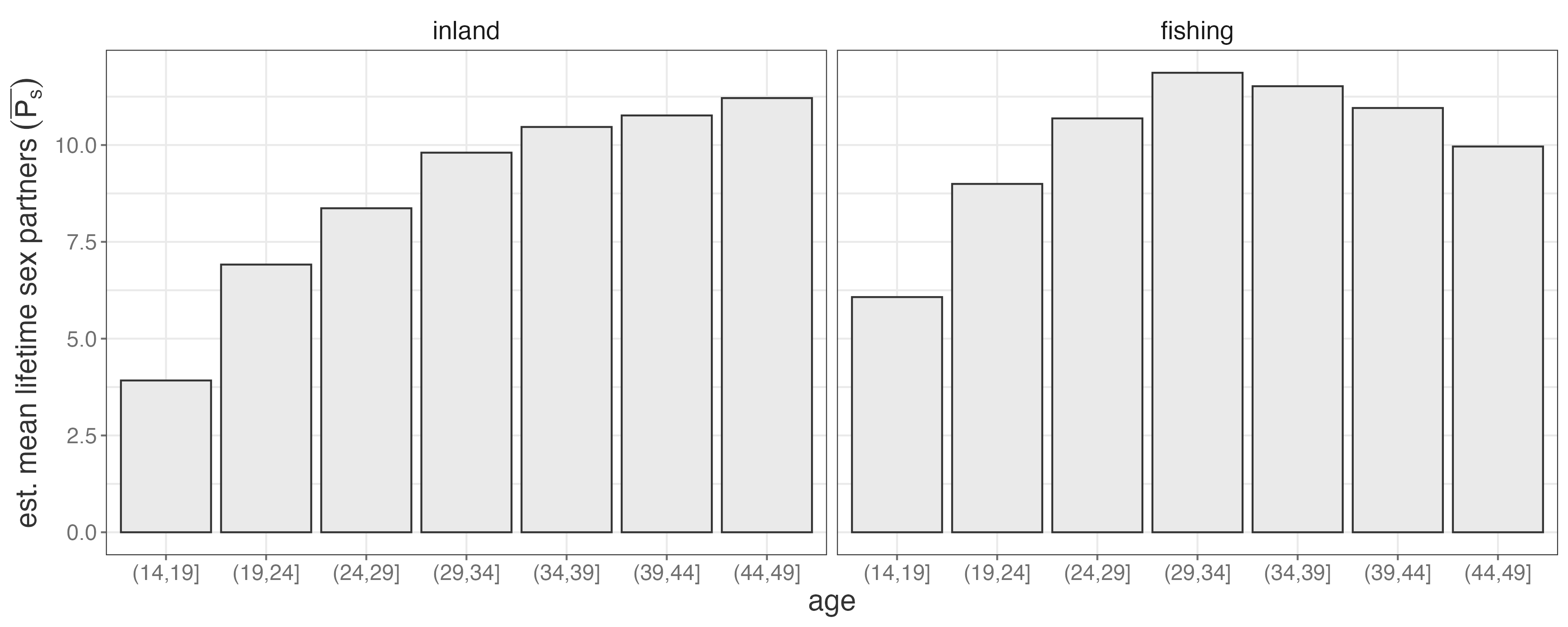

Supplement: S8 Fig — Includes simple imputation of categorical responses (e.g. “1-2” and “3+”) to 1) the mean value of observed responses of 1 or 2 (“1-2”) within age category and community type and 2) the mean of a lognormal distribution fit to observed responses of ≥ 3 lifetime sex partners within age category and community type. (TIF) [file ppat.1013065.s008.tif]

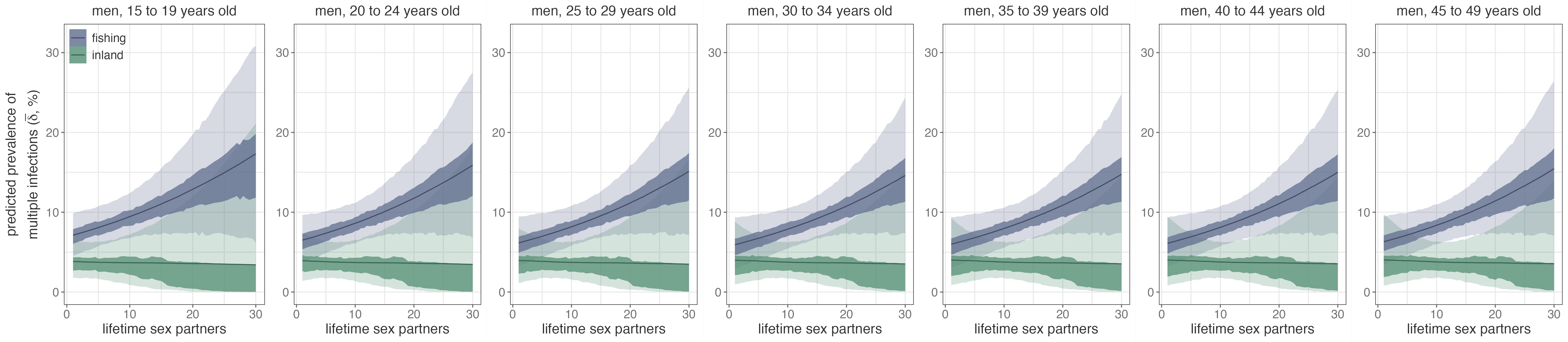

Supplement: S9 Fig — Median estimate is plotted as a line and shading represents the 50% and 95% highest posterior densities. All age categories share the same coefficient estimates but differ because lifetime sex partner values are standardized to the mean of the observed values within groups defined by sex, age category, and community type. (TIF) [file ppat.1013065.s009.tif]
