# Supplementary material for: Quantifying prevalence and risk factors of HIV multiple infection in Uganda from population-based deep-sequence data
Source: PLoS Pathog. 2025 Apr 22;21(4):e1013065. doi: 10.1371/journal.ppat.1013065 (PMC12055032; doi:10.1371/journal.ppat.1013065)
Supplement: S4 File — (PDF) [file ppat.1013065.s013.pdf]

# Quantifying prevalence and risk factors of HIV multiple infection in Uganda from population-based deep-sequence data

Supplementary File 4: Sensitivity of results to choice of genome windows

Michael A. Martin[1,*], Andrea Brizzi[2], Xiaoyue Xi[2,3], Ronald Moses Galiwango[4], Sikhulile Moyo[5,6], Deogratius Ssemwanga[7,8] Alexandra Blenkinsop[2], Andrew D. Redd[9,10,11], Lucie Abeler-Dörner[12], Christophe Fraser[12], Steven J. Reynolds[4,9,10], Thomas C. Quinn[4,9,10], Joseph Kagaayi[4,13], David Bonsall[14], David Serwadda[4], Gertrude Nakigozi[4], Godfrey Kigozi[4], M. Kate Grabowski[1,4,15,*], Oliver Ratmann[2,*], with the PANGEA-HIV Consortium and the Rakai Health Sciences Program

**1** Department of Pathology, Johns Hopkins School of Medicine, Baltimore, Maryland, United States of America
**2** Department of Mathematics, Imperial College London, London, United Kingdom
**3** Medical Research Council Biostatistics Unit, University of Cambridge, Cambridge, United Kingdom
**4** Rakai Health Sciences Program, Kalisizo, Uganda
**5** Botswana Harvard AIDS Institute Partnership, Botswana Harvard HIV Reference Laboratory, Gaborone, Botswana
**6** Harvard T.H. Chan School of Public Health, Boston, Massachusetts, United States of America
**7** Medical Research Council/Uganda Virus Research Institute and London School of Hygiene and Tropical Medicine Uganda Research Unit, Entebbe, Uganda
**8** Uganda Virus Research Institute, Entebbe, Uganda
**9** Department of Medicine, Johns Hopkins School of Medicine, Baltimore, Maryland, United States of America
**10** Division of Intramural Research, National Institute of Allergy and Infectious Diseases, National Institutes of Health, Bethesda, Maryland, United States of America
**11** Institute of Infectious Disease and Molecular Medicine, University of Cape Town, Cape Town, South Africa
**12** Pandemic Sciences Institute, Nuffield Department of Medicine, University of Oxford, Oxford, United Kingdom
**13** Makerere University School of Public Health, Kampala, Uganda
**14** Wellcome Centre for Human Genetics, Nuffield Department of Medicine, University of Oxford, Oxford, United Kingdom
**15** Department of Epidemiology, Johns Hopkins Bloomberg School of Public Health, Baltimore, Maryland, United States of America

[*] mmart108@jhmi.edu (MAM); mgrabow2@jhu.edu (MKG); oliver.ratmann@imperial.ac.uk (OR)

## 1 Choice of genome window

Phylogenetic trees from the HIV deep-sequence reads generated from 2,029 Rakai Community Cohort Study participants living with viremic HIV using *phyloscanner*. In total, phylogenetic trees were inferred using data from 287 250 base pair (bp) overlapping windows with a 25 bp step between windows. To generate a set of independent data points from each sequenced participant-visit we first downsampled the 287 overlapping windows to a set of 29 250 bp windows beginning at position 800 in the HXB2 (GenBank: K03455.1) genome. Summarized data in the form of the genome coverage in the phyloscanner output ($N_i^{obs}$) and the number of windows with multiple phylogenetic subgraphs ($MI_i^{obs}$) was highly correlated between the set of full windows and the set of non-overlapping windows (Pearson's $\rho = 1$, 0.97, respectively, Genome Window Fig S.G.1).

Additionally, we simulate synthetic amplicon data by selecting data from *phyloscanner* windows spanning the *p24* and *gp41* regions of the HIV genome. Specifically, we aim to generate data covering nucleotides 1,427 through 1,816 (*p24*) and 7,941 through 8,264 (*gp41*) of HXB2 based on the amplicons used for earlier studies of HIV multiple infection [1, 2, 3]. As these regions are larger than our 250 bp *phyloscanner* windows we first

take data from the fist and the last window that are entirely contained within each target region. Sequenced participant-visits were assigned $N_{i,r}^{\text{obs}} = 1$ (where $r$ indicates the amplicon region) in each region if $N_{i,w}^{\text{obs}} = 1$ for both of the selected windows in a given amplicon region. We set $MI_{i,r}^{\text{obs}} = 1$ if $MI_{i,w}^{\text{obs}} = 1$ in either of the selected windows spanning a given amplicon region.

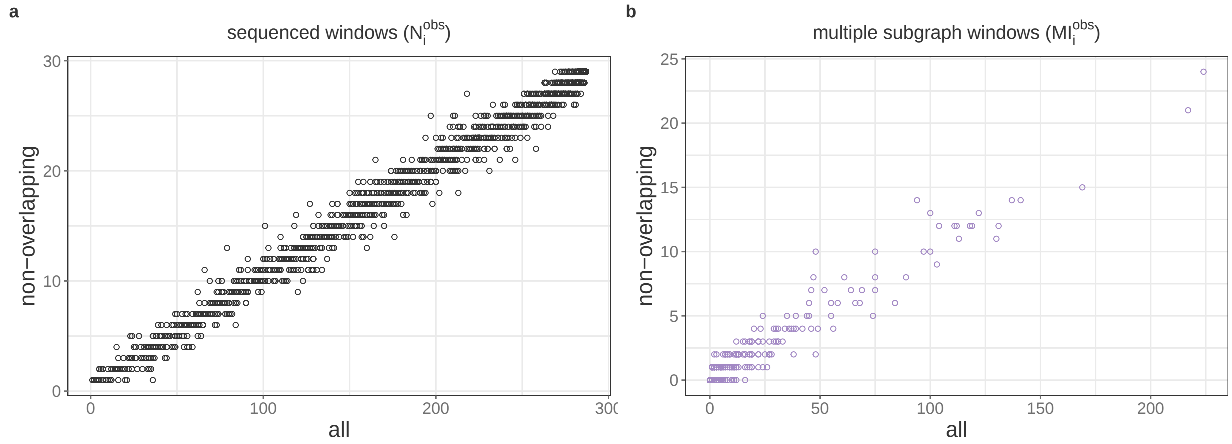


**Fig S.G.1. Correlation between summary statistics from within-host deep sequence phylogenies from all genome windows and non-overlapping windows generated from 2,029 Rakai Community Cohort Study living with viremic HIV.** (A) Number of windows with phyloscanner output for each participant-visit. (B) Number of windows with multiple phylogenetic subgraphs for each participant-visit.

# 2 Alternate set of non-overlapping windows

Based on the window size and window step size, there are 10 potential sets of non-overlapping windows that could have been chosen to generate a set of independent data-points for each sequenced participant visits. To assess the sensitivity of our results to the choice of non-overlapping windows we alternatively downsampled the data to the set of non-overlapping 250 bp windows beginning at position 950 in the HXB2 genome. Genome coverage and the number of windows with multiple phylogenetic subgraphs was highly correlated between the two sets of non-overlapping windows (Pearson's $\rho = 0.99$, 0.94, respectively).

# 3 Sensitivity of results to non-overlapping windows

Finally, we fit our inference model accounting for partial sequencing success, false negative multiple subgraph windows, and false positive multiple subgraph windows to data from the two sets of non-overlapping genome windows (Table S.G.1-S.G.2). We note that parameter estimates from the two model fits are highly similar, indicating that our results are not particularly sensitive to the exact choice of genome window sets.

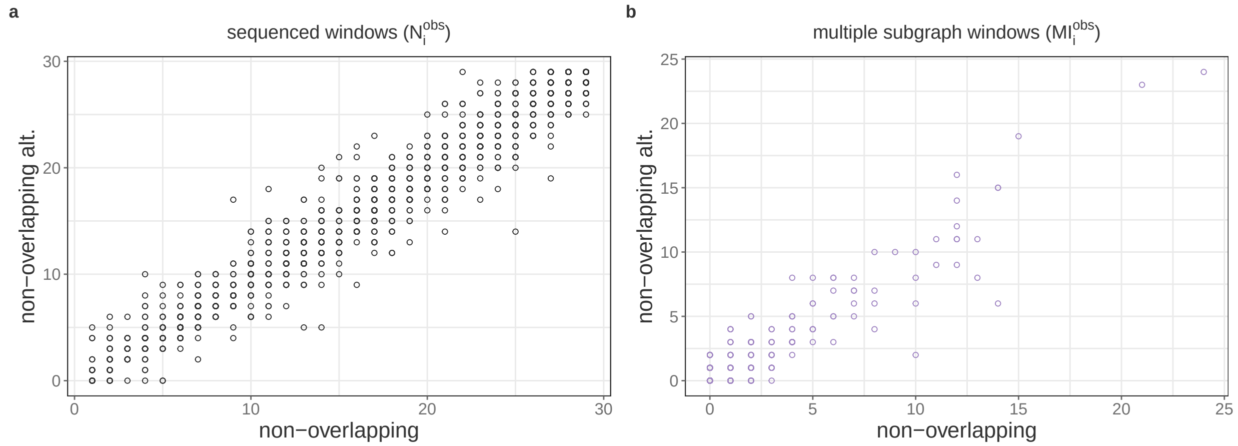


**Fig S.G.2. Correlation between summary statistics from within-host deep sequence phylogenies from all genome windows and non-overlapping windows generated from 2,029 Rakai Community Cohort Study participant-visits living with viremic HIV.** (A) Number of windows with phyloscanner output for each participant-visit. (B) Number of windows with multiple phylogenetic subgraphs for each participant-visit.

| Parameter | Prior | Median (95% HPD) | Bulk ESS | Tail ESS | $\hat{R}$ |
|---|---|---|---|---|---|
| $\alpha_0$ | Normal(0,$2^2$) | 1.22 (1.14, 1.29) | 856.49 | 1934.19 | 1 |
| $\alpha_1$ (amplicon) | $2 \times$ stz-MVN$_1(0,1)$ | -1.2 (-1.28, -1.12) | 720.29 | 1379.62 | 1 |
| $\alpha_2$ (bait-capture) | $2 \times$ stz-MVN$_1(0,1)$ | 1.2 (1.12, 1.28) | 720.29 | 1379.62 | 1 |
| $\alpha_3$ ($\log_{10}$ copies/mL) | Normal(0,$2^2$) | 1.19 (1.11, 1.27) | 789.99 | 1734.07 | 1 |
| $\alpha_4$ (amplicon $\times$ $\log_{10}$ copies/mL) | $2 \times$ stz-MVN$_2(0,1)$ | -0.27 (-0.35, -0.2) | 897.24 | 1860.66 | 1 |
| $\alpha_4$ (bait-capture $\times$ $\log_{10}$ copies/mL) | $2 \times$ stz-MVN$_2(0,1)$ | 0.27 (0.2, 0.35) | 897.24 | 1860.66 | 1 |
| $\sigma_\alpha$ | Half-Cauchy(0,1) | 1.52 (1.45, 1.59) | 2670.09 | 4963.67 | 1 |
| $\delta_0$ | Normal(0,$3.16^2$) | -3 (-3.31, -2.71) | 4053.65 | 5417.35 | 1 |
| $\beta_1$ ((14,24] years) | stz-MVN$_3(0,1)$ | -0.09 (-0.47, 0.29) | 6144.1 | 6019.96 | 1 |
| $\beta_2$ ((24,34] years)) | stz-MVN$_3(0,1)$ | 0 (-0.29, 0.31) | 7933.38 | 5636.94 | 1 |
| $\beta_3$ ((34,49] years) | stz-MVN$_3(0,1)$ | 0.09 (-0.24, 0.43) | 7392.71 | 6322.6 | 1 |
| $\beta_4$ (women) | stz-MVN$_4(0,1)$ | -0.12 (-0.34, 0.11) | 6887.8 | 5660.36 | 1 |
| $\beta_5$ (men) | stz-MVN$_4(0,1)$ | 0.12 (-0.11, 0.34) | 6887.8 | 5660.36 | 1 |
| $\beta_6$ (fishing) | stz-MVN$_5(0,1)$ | 0.44 (0.19, 0.72) | 6051.03 | 5610.97 | 1 |
| $\beta_7$ (inland) | stz-MVN$_5(0,1)$ | -0.44 (-0.72, -0.19) | 6051.03 | 5610.97 | 1 |
| logit($\lambda$) | Normal(0,1)[,2.2] | 0.31 (0.13, 0.49) | 3111.48 | 4673.84 | 1 |
| logit($\epsilon$) | Normal(0,1) | -5.73 (-5.95, -5.5) | 3430.6 | 4372.27 | 1 |

**Table S.G.1. Parameter estimates for full model fit to deep-sequence data from 2,029 RCCS participants living with viremic HIV with age, sex, and community type as putative risk factors for harboring multiple infections.** ESS = effective sample size. HPD = highest posterior density. stz-MVN = sum-to-zero multivariate-Normal distribution.

| Parameter | Prior | Median (95% HPD) | Bulk ESS | Tail ESS | $\hat{R}$ |
|---|---|---|---|---|---|
| $\alpha_0$ | Normal(0,$2^2$) | 1.2 (1.12, 1.28) | 664.44 | 1417.75 | 1 |
| $\alpha_1$ (amplicon) | $2 \times$ stz-MVN$_1(0,1)$ | -1.25 (-1.33, -1.17) | 658.4 | 1466.37 | 1 |
| $\alpha_2$ (bait-capture) | $2 \times$ stz-MVN$_1(0,1)$ | 1.25 (1.17, 1.33) | 658.4 | 1466.37 | 1 |
| $\alpha_3$ ($\log_{10}$ copies/mL) | Normal(0,$2^2$) | 1.19 (1.1, 1.27) | 814.03 | 1926.08 | 1 |
| $\alpha_4$ (amplicon $\times$ $\log_{10}$ copies/mL) | $2 \times$ stz-MVN$_2(0,1)$ | -0.28 (-0.36, -0.2) | 690.26 | 1797.25 | 1 |
| $\alpha_4$ (bait-capture $\times$ $\log_{10}$ copies/mL) | $2 \times$ stz-MVN$_2(0,1)$ | 0.28 (0.2, 0.36) | 690.26 | 1797.25 | 1 |
| $\sigma_\alpha$ | Half-Cauchy(0,1) | 1.51 (1.44, 1.57) | 2397.03 | 4039.68 | 1 |
| $\delta_0$ | Normal(0,$3.16^2$) | -2.91 (-3.19, -2.64) | 4365.06 | 4395.5 | 1 |
| $\beta_1$ ((14,24] years) | stz-MVN$_3(0,1)$ | -0.03 (-0.43, 0.32) | 5706.65 | 5511.77 | 1 |
| $\beta_2$ ((24,34] years)) | stz-MVN$_3(0,1)$ | -0.05 (-0.34, 0.25) | 8368.18 | 6439.92 | 1 |
| $\beta_3$ ((34,49] years) | stz-MVN$_3(0,1)$ | 0.08 (-0.26, 0.41) | 7309.88 | 6295.84 | 1 |
| $\beta_4$ (women) | stz-MVN$_4(0,1)$ | -0.05 (-0.28, 0.19) | 6175.95 | 5928.48 | 1 |
| $\beta_5$ (men) | stz-MVN$_4(0,1)$ | 0.05 (-0.19, 0.28) | 6175.95 | 5928.48 | 1 |
| $\beta_6$ (fishing) | stz-MVN$_5(0,1)$ | 0.38 (0.13, 0.64) | 6103.59 | 5919.35 | 1 |
| $\beta_7$ (inland) | stz-MVN$_5(0,1)$ | -0.38 (-0.64, -0.13) | 6103.59 | 5919.35 | 1 |
| logit($\lambda$) | Normal(0,1)[,2.2] | 0.49 (0.33, 0.64) | 3958.3 | 5424.11 | 1 |
| logit($\epsilon$) | Normal(0,1) | -5.86 (-6.11, -5.61) | 3245.91 | 5023.33 | 1 |

**Table S.G.2. Parameter estimates for full model fit to deep-sequence data from 2,029 RCCS participants living with viremic HIV with age, sex, and community type as putative risk factors for harboring multiple infections using alternative non-overlapping windows.** ESS = effective sample size. HPD = highest posterior density. stz-MVN = sum-to-zero multivariate-Normal distribution.

# References


[1] Redd AD, Collinson-Streng A, Martens C, Ricklefs S, Mullis CE, Manucci J, et al. Identification of HIV Superinfection in Seroconcordant Couples in Rakai, Uganda, by Use of Next-Generation Deep Sequencing. Journal of Clinical Microbiology. 2011;49(8):2859–2867. doi:10.1128/jcm.00804-11.

[2] Redd AD, Mullis CE, Serwadda D, Kong X, Martens C, Ricklefs SM, et al. The Rates of HIV Superinfection and Primary HIV Incidence in a General Population in Rakai, Uganda. The Journal of Infectious Diseases. 2012;206(2):267–274. doi:10.1093/infdis/jis325.

[3] Redd AD, Ssemwanga D, Vandepitte J, Wendel SK, Ndembi N, Bukenya J, et al. Rates of HIV-1 superinfection and primary HIV-1 infection are similar in female sex workers in Uganda. AIDS. 2014;28(14):2147–2152. doi:10.1097/QAD.0000000000000365.
