# Supplementary material for: Quantifying prevalence and risk factors of HIV multiple infection in Uganda from population-based deep-sequence data
Source: PLoS Pathog. 2025 Apr 22;21(4):e1013065. doi: 10.1371/journal.ppat.1013065 (PMC12055032; doi:10.1371/journal.ppat.1013065)
Supplement: S1 Table — (PDF) [file ppat.1013065.s014.pdf]

	Participants	
Survey round	amplicon (PANGEA1)	bait-capture (PANGEA2)
2010	0	16
2012	720	29
2014	296	50
2015	14	509
2017	0	328
2019	0	67
