# Supplementary material for: Quantifying prevalence and risk factors of HIV multiple infection in Uganda from population-based deep-sequence data
Source: PLoS Pathog. 2025 Apr 22;21(4):e1013065. doi: 10.1371/journal.ppat.1013065 (PMC12055032; doi:10.1371/journal.ppat.1013065)
Supplement: S2 Table — For each participant, includes data from the participant-visit processed with PHSC if applicable or the participant-visit with the highest viral load, using the first visit in the case of ties or for people not living with HIV. Percentages represent the row percentages within each category. Binomial confidence intervals were calculated using the Agresti–Coull method. PHSC = phyloscanner. (PDF) [file ppat.1013065.s015.pdf]

	Participants		
		Living w/ HIV (%)	
	All	n (%)	% of All
Overall	50,967	8,841	19,503
Survey Round			
2010	15,663 (30.73%)	1,812 (21.72%)	11.57% (11.08% - 12.08%)
2012	9,985 (19.59%)	2,202 (26.4%)	22.05% (21.25% - 22.88%)
2014	7,032 (13.8%)	1,170 (14.03%)	16.64% (15.79% - 17.53%)
2015	6,364 (12.49%)	1,292 (15.49%)	20.3% (19.33% - 21.31%)
2017	6,158 (12.08%)	1,080 (12.95%)	17.54% (16.61% - 18.51%)
2019	5,765 (11.31%)	786 (9.42%)	13.63% (12.77% - 14.54%)
Sex			
Female	27,621 (54.19%)	5,315 (63.71%)	19.24% (18.78% - 19.71%)
Male	23,346 (45.81%)	3,027 (36.29%)	12.97% (12.54% - 13.4%)
Commiunity type			
Inland	41,508 (81.44%)	4,974 (59.63%)	11.98% (11.67% - 12.3%)
Fishing	9,459 (18.56%)	3,368 (40.37%)	35.61% (34.65% - 36.58%)
Age			
(14, 24]	24,356 (47.79%)	1,472 (17.65%)	6.04% (5.75% - 6.35%)
(24, 34]	24,356 (31.38%)	3,875 (46.45%)	24.23% (23.57% - 24.9%)
(34, 49]	10,616 (20.83%)	2,995 (35.9%)	28.21% (27.36% - 29.08%)
