# Supplementary material for: Quantifying prevalence and risk factors of HIV multiple infection in Uganda from population-based deep-sequence data
Source: PLoS Pathog. 2025 Apr 22;21(4):e1013065. doi: 10.1371/journal.ppat.1013065 (PMC12055032; doi:10.1371/journal.ppat.1013065)
Supplement: S3 Table — For each participant, includes data from the participant-visit processed with PHSC if applicable or the participant-visit with the highest viral load, using the first visit in the case of ties or for people not living with HIV. In each category the percentage represents the percentage of all participants or all participants that were viremic and processed with PHSC. (PDF) [file ppat.1013065.s016.pdf]

Variable	Participant-visits with missing data (%)	
	All (%)	Viremic & Processed w PHSC (%)
HIV serostatus	104 (0.2%)	0 (0%)
Sex	0 (0%)	0 (0%)
Age	0 (0%)	0 (0%)
Circumcision	5791 (11.36%)	54 (2.66%)
Community type	0 (0%)	0 (0%)
Married	7 (0.01%)	0 (0%)
Lifetime sex partners	0 (0%)	0 (0%)
In-migrant	179 (0.35%)	5 (0.25%)
Sex & bar/restaurant work	27 (0.05%)	0 (0%)
