# Supplementary material for: Quantifying prevalence and risk factors of HIV multiple infection in Uganda from population-based deep-sequence data
Source: PLoS Pathog. 2025 Apr 22;21(4):e1013065. doi: 10.1371/journal.ppat.1013065 (PMC12055032; doi:10.1371/journal.ppat.1013065)
Supplement: S4 Table — Epidemiological strata are defined by community type, age category, and sex. As viral load testing was not routinely conducted in earlier study rounds, the viremic participants belonging to each strata were tabulated using only data from the 2014 through 2019 surveys. (PDF) [file ppat.1013065.s017.pdf]

Age	Sex	Community type	W_s, Participant-visits 2014 - 2019 (%)	Q_s, Participants w/ PHSC (%)
(14, 24]	F	Inland	12018 (15.71%)	118 (5.82%)
(14, 24]	F	Fishing	2349 (3.07%)	195 (9.61%)
(14, 24]	M	Inland	10537 (13.77%)	37 (1.82%)
(14, 24]	M	Fishing	1986 (2.6%)	81 (3.99%)
(24, 34]	F	Inland	10523 (13.76%)	209 (10.3%)
(24, 34]	F	Fishing	3416 (4.47%)	312 (15.38%)
(24, 34]	M	Inland	7454 (9.74%)	165 (8.13%)
(24, 34]	M	Fishing	3625 (4.74%)	366 (18.04%)
(34, 49]	F	Inland	10473 (13.69%)	90 (4.44%)
(34, 49]	F	Fishing	2373 (3.1%)	108 (5.32%)
(34, 49]	M	Inland	8474 (11.08%)	123 (6.06%)
(34, 49]	M	Fishing	3273 (4.28%)	225 (11.09%)
