# Supplementary material for: Quantifying prevalence and risk factors of HIV multiple infection in Uganda from population-based deep-sequence data
Source: PLoS Pathog. 2025 Apr 22;21(4):e1013065. doi: 10.1371/journal.ppat.1013065 (PMC12055032; doi:10.1371/journal.ppat.1013065)
Supplement: S5 Table — ESS = effective sample size. HPD = highest posterior density. (PDF) [file ppat.1013065.s018.pdf]

Parameter	True Value	Prior	Median (95% HPD)	Bulk ESS	Tail ESS	$\hat{R}$
α_0	2	Normal(0,2^2)	1.99 (1.93, 2.05)	1825.16	2696.13	1
α_1	2	Normal(0,2^2)	2.02 (1.95, 2.09)	1640.83	3180.42	1
σ_{ind}	1	Half-Cauchy(0,1)	1 (0.95, 1.05)	2214	3845.32	1
δ_0	-2.94	Normal(0,3.16^2)	-2.92 (-3.13, -2.71)	9297.7	5380.48	1
