# Supplementary material for: Quantifying prevalence and risk factors of HIV multiple infection in Uganda from population-based deep-sequence data
Source: PLoS Pathog. 2025 Apr 22;21(4):e1013065. doi: 10.1371/journal.ppat.1013065 (PMC12055032; doi:10.1371/journal.ppat.1013065)
Supplement: S6 Table — ESS = effective sample size. HPD = highest posterior density. (PDF) [file ppat.1013065.s019.pdf]

Parameter	True Value	Prior	Median (95% HPD)	Bulk ESS	Tail ESS	$\hat{R}$
α_0	2	Normal(0,2^2)	1.2 (1.13, 1.28)	958.69	1904.79	1
α_1	2	Normal(0,2^2)	1.39 (1.31, 1.47)	1073.17	2266.37	1
σ_{ind}	1	Half-Cauchy(0,1)	1.51 (1.45, 1.57)	4099.54	4610.11	1
δ_0	-2.94	Normal(0,3.16^2)	-1.02 (-1.12, -0.92)	13116.56	4937.73	1
