# Supplementary material for: Quantifying prevalence and risk factors of HIV multiple infection in Uganda from population-based deep-sequence data
Source: PLoS Pathog. 2025 Apr 22;21(4):e1013065. doi: 10.1371/journal.ppat.1013065 (PMC12055032; doi:10.1371/journal.ppat.1013065)
Supplement: S7 Table — ESS = effective sample size. HPD = highest posterior density. (PDF) [file ppat.1013065.s020.pdf]

| Parameter | True Value | Prior | Median (95% HPD) | Bulk ESS | Tail ESS | $\hat{R}$ |
|---|---|---|---|---|---|---|
| $\alpha_0$ | 2 | Normal(0,$2^2$) | 1.99 (1.93, 2.05) | 4202.98 | 6050.69 | 1 |
| $\alpha_1$ | 2 | Normal(0,$2^2$) | 2.03 (1.96, 2.1) | 4512.33 | 5180.9 | 1 |
| $\sigma_{ind}$ | 1 | Half-Cauchy(0,1) | 1 (0.95, 1.05) | 3415.45 | 5123.79 | 1 |
| $\delta_0$ | -2.94 | Normal(0,$3.16^2$) | -2.91 (-3.12, -2.7) | 13924.79 | 4653.77 | 1 |
| logit($\lambda$) | -0.85 | Normal(0,1) | -0.76 (-0.88, -0.63) | 12502.11 | 6193.21 | 1 |
| logit($\epsilon$) | -4.6 | Normal(0,1) | -4.51 (-4.6, -4.41) | 17064.32 | 5663.25 | 1 |
