# Supplementary material for: Quantifying prevalence and risk factors of HIV multiple infection in Uganda from population-based deep-sequence data
Source: PLoS Pathog. 2025 Apr 22;21(4):e1013065. doi: 10.1371/journal.ppat.1013065 (PMC12055032; doi:10.1371/journal.ppat.1013065)
Supplement: S8 Table — ESS = effective sample size. HPD = highest posterior density. stz-MVN = sum-to-zero multivariate Normal distribution. (PDF) [file ppat.1013065.s021.pdf]

Parameter	True Value	Prior	Median (95% HPD)	Bulk ESS	Tail ESS	$\hat{R}$
α_0	2	Normal(0,2^2)	2 (1.94, 2.06)	2538.05	4223.07	1
α_1	2	Normal(0,2^2)	1.99 (1.92, 2.07)	2734.33	4442.8	1
σ_{ind}	1	Half-Cauchy(0,1)	0.99 (0.94, 1.04)	2940.12	4996.89	1
δ_0	-2.94	Normal(0,3.16^2)	-2.58 (-2.76, -2.38)	12220.09	5929.73	1
logit(λ)	-0.85	Normal(0,1)	-0.72 (-0.81, -0.62)	10214.88	5691.82	1
logit(ϵ)	-4.6	Normal(0,1)	-4.55 (-4.65, -4.46)	11950.2	5981.25	1
β_1	-0.37	$\tau \times$ stz-MVN$_1(0, \xi_j^2)$	-0.3 (-0.52, -0.09)	5622.29	3474.6	1
β_2	0.37	$\tau \times$ stz-MVN$_1(0, \xi_j^2)$	0.3 (0.09, 0.52)	5622.29	3474.6	1
β_3	0	$\tau \times$ stz-MVN$_2(0, \xi_j^2)$	0 (-0.13, 0.13)	9807.47	7764.69	1
β_4	0	$\tau \times$ stz-MVN$_2(0, \xi_j^2)$	0 (-0.13, 0.13)	9807.47	7764.69	1
β_5	0	$\tau \times$ stz-MVN$_3(0, \xi_j^2)$	0.04 (-0.08, 0.21)	6381.25	6547.65	1
β_6	0	$\tau \times$ stz-MVN$_3(0, \xi_j^2)$	-0.04 (-0.21, 0.08)	6381.25	6547.65	1
β_7	0	$\tau \times$ stz-MVN$_4(0, \xi_j^2)$	0 (-0.12, 0.14)	10368.98	7768.22	1
β_8	0	$\tau \times$ stz-MVN$_4(0, \xi_j^2)$	0 (-0.14, 0.12)	10368.98	7768.22	1
β_9	0	$\tau \times$ stz-MVN$_5(0, \xi_j^2)$	0.02 (-0.1, 0.17)	8136.51	7486.64	1
β_{10}	0	$\tau \times$ stz-MVN$_5(0, \xi_j^2)$	-0.02 (-0.17, 0.1)	8136.51	7486.64	1
τ	-	Half-Cauchy(0,1)	0.25 (0.01, 0.8)	4553.02	5177.89	1
ξ_1	-	Half-$t_2(0, 1)$	1.29 (0.02, 5.33)	6851.93	5440.52	1
ξ_2	-	Half-$t_2(0, 1)$	1.27 (0.07, 5.33)	7520.04	5456.36	1
ξ_3	-	Half-$t_2(0, 1)$	0.57 (0, 3.45)	4886.47	4398.78	1
ξ_4	-	Half-$t_2(0, 1)$	0.56 (0, 3.62)	4607.39	4237.79	1
ξ_5	-	Half-$t_2(0, 1)$	0.65 (0, 3.71)	5269.38	4521.63	1
ξ_6	-	Half-$t_2(0, 1)$	0.63 (0, 3.67)	4695.61	4525.06	1
ξ_7	-	Half-$t_2(0, 1)$	0.56 (0, 3.45)	4392.15	4203.77	1
ξ_8	-	Half-$t_2(0, 1)$	0.57 (0, 3.49)	5168.07	4580.58	1
ξ_9	-	Half-$t_2(0, 1)$	0.59 (0, 3.65)	5418.99	4681.89	1
ξ_{10}	-	Half-$t_2(0, 1)$	0.59 (0, 3.59)	4799.55	4411.37	1
