# Supplementary material for: Quantifying prevalence and risk factors of HIV multiple infection in Uganda from population-based deep-sequence data
Source: PLoS Pathog. 2025 Apr 22;21(4):e1013065. doi: 10.1371/journal.ppat.1013065 (PMC12055032; doi:10.1371/journal.ppat.1013065)
Supplement: S9 Table — ESS = effective sample size. HPD = highest posterior density. stz-MVN = sum-to-zero multivariate Normal distribution. (PDF) [file ppat.1013065.s022.pdf]

Parameter	Prior	Median (95% HPD)	Bulk ESS	Tail ESS	$\hat{R}$
α_0	Normal(0,2^2)	1.22 (1.14, 1.29)	856.49	1934.19	1
α_1 (amplicon)	$2 \times$ stz-MVN$_1(0,1)$	-1.2 (-1.28, -1.12)	720.29	1379.62	1
α_2 (bait-capture)	$2 \times$ stz-MVN$_1(0,1)$	1.2 (1.12, 1.28)	720.29	1379.62	1
α_3 ($\log_{10}$ copies/mL)	Normal(0,2^2)	1.19 (1.11, 1.27)	789.99	1734.07	1
α_4 (amplicon $\times$ $\log_{10}$ copies/mL)	$2 \times$ stz-MVN$_2(0,1)$	-0.27 (-0.35, -0.2)	897.24	1860.66	1
α_5 (bait-capture $\times$ $\log_{10}$ copies/mL)	$2 \times$ stz-MVN$_2(0,1)$	0.27 (0.2, 0.35)	897.24	1860.66	1
σ_{ind}	Half-Cauchy(0,1)	1.52 (1.45, 1.59)	2670.09	4963.67	1
δ_0	Normal(0,3.16^2)	-3 (-3.31, -2.71)	4053.65	5417.35	1
β_1 ((14,24] years)	stz-MVN$_3(0,1)$	-0.09 (-0.47, 0.29)	6144.1	6019.96	1
β_2 ((24,34] years)	stz-MVN$_3(0,1)$	0 (-0.29, 0.31)	7933.38	5636.94	1
β_3 ((34,49] years))	stz-MVN$_3(0,1)$	0 (-0.29, 0.31)	7933.38	5636.94	1
β_4 (women)	stz-MVN$_4(0,1)$	-0.12 (-0.34, 0.11)	6887.8	5660.36	1
β_5 (men)	stz-MVN$_4(0,1)$	0.12 (-0.11, 0.34)	6887.8	5660.36	1
β_6 (fishing)	stz-MVN$_5(0,1)$	0.44 (0.19, 0.72)	6051.03	5610.97	1
β_7 (inland)	stz-MVN$_5(0,1)$	-0.44 (-0.72, -0.19)	6051.03	5610.97	1
logit(λ)	Normal(0,1)[,2.2]	0.31 (0.13, 0.49)	3111.48	4673.84	1
logit(ϵ)	Normal(0,1)	-5.73 (-5.95, -5.5)	3430.6	4372.27	1

Parameter estimates for full model fit to deep-sequence data from 2,029 RCCS participants living with viremic HIV with age, sex, and community type as putative risk factors for harboring multiple infections ESS = effective sample size. HPD = highest posterior density. stz-MVN = sum-to-zero multivariate Normal distribution.
