# Supplementary material for: Quantifying prevalence and risk factors of HIV multiple infection in Uganda from population-based deep-sequence data
Source: PLoS Pathog. 2025 Apr 22;21(4):e1013065. doi: 10.1371/journal.ppat.1013065 (PMC12055032; doi:10.1371/journal.ppat.1013065)
Supplement: S10 Table — Includes minor subgraphs supported in < 1% of reads in a given window so long as they are supported by at least three reads. ESS = effective sample size. HPD = highest posterior density. stz-MVN = sum-to-zero multivariate Normal distribution. (PDF) [file ppat.1013065.s023.pdf]

Parameter	Prior	Median (95% HPD)	Bulk ESS	Tail ESS	$\hat{R}$
α_0	Normal(0,2^2)	1.21 (1.14, 1.3)	715.95	1606.76	1
α_1 (amplicon)	$2 \times$ stz-$\text{MVN}_1(0,1)$	-1.2 (-1.28, -1.13)	575.57	992.64	1.01
α_2 (bait-capture)	$2 \times$ stz-$\text{MVN}_1(0,1)$	1.2 (1.13, 1.28)	575.57	992.64	1.01
α_3 ($\log_{10}$ copies/mL)	Normal(0,2^2)	1.19 (1.11, 1.27)	690.77	1594.82	1
α_4 (amplicon $\times$ $\log_{10}$ copies/mL)	$2 \times$ stz-$\text{MVN}_2(0,1)$	-0.28 (-0.36, -0.2)	680.53	1225.86	1
α_5 (bait-capture $\times$ $\log_{10}$ copies/mL)	$2 \times$ stz-$\text{MVN}_2(0,1)$	0.28 (0.2, 0.36)	680.53	1225.86	1
σ_{ind}	Half-Cauchy(0,1)	1.51 (1.45, 1.58)	2291.15	4077.76	1
δ_0	Normal(0,3.16^2)	-2.98 (-3.26, -2.68)	3490.63	5027.37	1
β_1 ((14,24] years)	stz-$\text{MVN}_3(0,1)$	-0.07 (-0.45, 0.3)	4543.73	4783.69	1
β_2 ((24,34] years)	stz-$\text{MVN}_3(0,1)$	0.02 (-0.27, 0.31)	6648.23	5357.19	1
β_3 ((34,49] years))	stz-$\text{MVN}_3(0,1)$	0.06 (-0.28, 0.39)	4744.34	5520.46	1
β_4 (women)	stz-$\text{MVN}_4(0,1)$	-0.08 (-0.32, 0.13)	4909.37	5316.81	1
β_5 (men)	stz-$\text{MVN}_4(0,1)$	0.08 (-0.13, 0.32)	4909.37	5316.81	1
β_6 (fishing)	stz-$\text{MVN}_5(0,1)$	0.46 (0.21, 0.74)	4270.02	4902.38	1
β_7 (inland)	stz-$\text{MVN}_5(0,1)$	-0.46 (-0.74, -0.21)	4270.02	4902.38	1
logit(λ)	Normal(0,1)[,2.2]	0.31 (0.13, 0.47)	3323.97	4637.46	1
logit(ϵ)	Normal(0,1)	-5.68 (-5.91, -5.46)	3087.78	4832.41	1
