# Supplementary material for: Quantifying prevalence and risk factors of HIV multiple infection in Uganda from population-based deep-sequence data
Source: PLoS Pathog. 2025 Apr 22;21(4):e1013065. doi: 10.1371/journal.ppat.1013065 (PMC12055032; doi:10.1371/journal.ppat.1013065)
Supplement: S11 Table — Includes data from genome windows spanning the p24 (1427–1816) and gp41 (7941–8264) regions. ESS = effective sample size. HPD = highest posterior density. stz-MVN = sum-to-zero multivariate Normal distribution. (PDF) [file ppat.1013065.s024.pdf]

Parameter	Prior	Median (95% HPD)	Bulk ESS	Tail ESS	$\hat{R}$
α_0	Normal(0,2^2)	0.96 (0.8, 1.12)	10807.93	6207.49	1
α_1 (amplicon)	$2 \times$ stz-MVN$_1(0,1)$	-1.45 (-1.6, -1.31)	10792.96	6645.25	1
α_2 (bait-capture)	$2 \times$ stz-MVN$_1(0,1)$	1.45 (1.31, 1.6)	10792.96	6645.25	1
α_3 ($\log_{10}$ copies/mL)	Normal(0,2^2)	1.23 (1.07, 1.4)	10569.9	6755.2	1
α_4 (amplicon $\times$ $\log_{10}$ copies/mL)	$2 \times$ stz-MVN$_2(0,1)$	-0.14 (-0.3, 0.01)	9687.87	6219.33	1
α_5 (bait-capture $\times$ $\log_{10}$ copies/mL)	$2 \times$ stz-MVN$_2(0,1)$	0.14 (-0.01, 0.3)	9687.87	6219.33	1
δ_0	Normal(0,3.16^2)	-4.53 (-5.92, -3.39)	5207.26	4974.06	1
β_1 ((14,24] years)	stz-MVN$_3(0,1)$	0.26 (-0.71, 1.26)	10055.64	4979.66	1
β_2 ((24,34] years)	stz-MVN$_3(0,1)$	0.25 (-0.6, 1.13)	11967.57	5302.78	1
β_3 ((34,49] years))	stz-MVN$_3(0,1)$	-0.46 (-1.68, 0.52)	10950.57	5397.65	1
β_4 (women)	stz-MVN$_4(0,1)$	-0.11 (-0.78, 0.61)	12335.07	5032.56	1
β_5 (men)	stz-MVN$_4(0,1)$	0.11 (-0.61, 0.78)	12335.07	5032.56	1
β_6 (fishing)	stz-MVN$_5(0,1)$	0.82 (0.01, 1.86)	8852.47	4584.36	1
β_7 (inland)	stz-MVN$_5(0,1)$	-0.82 (-1.86, -0.01)	8852.47	4584.36	1
logit(λ)	Normal(0,1)[,2.2]	-0.86 (-2.35, 0.46)	10026.92	5401.82	1
logit(ϵ)	Normal(0,1)	-3.74 (-4.06, -3.43)	10702.39	5970.78	1
