# Supplementary material for: Quantifying prevalence and risk factors of HIV multiple infection in Uganda from population-based deep-sequence data
Source: PLoS Pathog. 2025 Apr 22;21(4):e1013065. doi: 10.1371/journal.ppat.1013065 (PMC12055032; doi:10.1371/journal.ppat.1013065)
Supplement: S12 Table — ESS = effective sample size. HPD = highest posterior density. stz-MVN = sum-to-zero multivariate Normal distribution. (PDF) [file ppat.1013065.s025.pdf]

Parameter	Prior	Median (95% HPD)	Bulk ESS	Tail ESS	$\hat{R}$
α_0	Normal(0,2^2)	1.21 (1.14, 1.29)	784.08	1779.86	1
α_1 (amplicon)	$2 \times$ stz-MVN$_1(0,1)$	-1.21 (-1.29, -1.13)	669.69	1728.68	1.01
α_2 (bait-capture)	$2 \times$ stz-MVN$_1(0,1)$	1.21 (1.13, 1.29)	669.69	1728.68	1.01
α_3 ($\log_{10}$ copies/mL)	Normal(0,2^2)	1.19 (1.11, 1.28)	611.57	1363.09	1
α_4 (amplicon $\times$ $\log_{10}$ copies/mL)	$2 \times$ stz-MVN$_2(0,1)$	-0.27 (-0.35, -0.2)	794.75	1350.48	1
α_5 (bait-capture $\times$ $\log_{10}$ copies/mL)	$2 \times$ stz-MVN$_2(0,1)$	0.27 (0.2, 0.35)	794.75	1350.48	1
σ_{ind}	Half-Cauchy(0,1)	1.52 (1.46, 1.59)	2846.06	4329.07	1
δ_0	Normal(0,3.16^2)	-2.77 (-2.99, -2.53)	4117.15	4922.89	1
β_1 (amplicon)	stz-MVN$_3(0,1)$	0.23 (0.02, 0.46)	6679.26	5445.09	1
β_2 (bait-capture)	stz-MVN$_3(0,1)$	-0.23 (-0.46, -0.02)	6679.26	5445.09	1
logit(λ)	Normal(0,1)[,2.2]	0.29 (0.11, 0.46)	2899.52	5113.97	1
logit(ϵ)	Normal(0,1)	-5.73 (-5.97, -5.51)	3017.4	4579.35	1
