# Supplementary material for: Quantifying prevalence and risk factors of HIV multiple infection in Uganda from population-based deep-sequence data
Source: PLoS Pathog. 2025 Apr 22;21(4):e1013065. doi: 10.1371/journal.ppat.1013065 (PMC12055032; doi:10.1371/journal.ppat.1013065)
Supplement: S13 Table — ESS = effective sample size. HPD = highest posterior density. stz-MVN = sum-to-zero multivariate Normal distribution. (PDF) [file ppat.1013065.s026.pdf]

Parameter	Prior	Median (95% HPD)	Bulk ESS	Tail ESS	$\hat{R}$
α_0	Normal(0,2^2)	1.21 (1.14, 1.29)	1014.37	1840.94	1
α_1 (amplicon)	$2 \times$ stz-MVN$_1(0,1)$	-1.21 (-1.29, -1.13)	869.84	1256.69	1
α_2 (bait-capture)	$2 \times$ stz-MVN$_1(0,1)$	1.21 (1.13, 1.29)	869.84	1256.69	1
α_3 ($\log_{10}$ copies/mL)	Normal(0,2^2)	1.19 (1.11, 1.27)	955.33	2001.4	1
α_4 (amplicon $\times$ $\log_{10}$ copies/mL)	$2 \times$ stz-MVN$_2(0,1)$	-0.27 (-0.35, -0.2)	956.01	2054.49	1
α_5 (bait-capture $\times$ $\log_{10}$ copies/mL)	$2 \times$ stz-MVN$_2(0,1)$	0.27 (0.2, 0.35)	956.01	2054.49	1
σ_{ind}	Half-Cauchy(0,1)	1.52 (1.45, 1.58)	2717.44	4514.42	1
δ_0	Normal(0,3.16^2)	-2.94 (-3.22, -2.66)	4147.97	5141.96	1
β_1 (fishing)	stz-MVN$_3(0,1)$	0.41 (0.14, 0.69)	5017.48	5234.63	1
β_2 (inland)	stz-MVN$_3(0,1)$	-0.41 (-0.69, -0.14)	5017.48	5234.63	1
β_3 (amplicon)	stz-MVN$_4(0,1)$	0.14 (-0.08, 0.37)	6583.04	6017.95	1
β_4 (bait-capture)	stz-MVN$_4(0,1)$	-0.14 (-0.37, 0.08)	6583.04	6017.95	1
logit(λ)	Normal(0,1)[,2.2]	0.3 (0.12, 0.47)	3592.02	4732.85	1
logit(ϵ)	Normal(0,1)	-5.73 (-5.96, -5.5)	3567.6	4924.73	1
