# Supplementary material for: Quantifying prevalence and risk factors of HIV multiple infection in Uganda from population-based deep-sequence data
Source: PLoS Pathog. 2025 Apr 22;21(4):e1013065. doi: 10.1371/journal.ppat.1013065 (PMC12055032; doi:10.1371/journal.ppat.1013065)
Supplement: S14 Table — ESS = effective sample size. HPD = highest posterior density. stz-MVN = sum-to-zero multivariate Normal distribution. (PDF) [file ppat.1013065.s027.pdf]

Parameter	Prior	Median (95% HPD)	Bulk ESS	Tail ESS	$\hat{R}$
α_0	Normal(0,2^2)	1.43 (1.31, 1.55)	681.22	1862.36	1
α_1 (amplicon)	$2 \times$ stz-MVN$_1(0, 1)$	-1.26 (-1.37, -1.15)	491.8	1169.81	1
α_2 (bait-capture)	$2 \times$ stz-MVN$_1(0, 1)$	1.26 (1.15, 1.37)	491.8	1169.81	1
α_3 ($\log_{10}$ copies/mL)	Normal(0,2^2)	1.17 (1.05, 1.3)	843.93	1877.54	1
α_4 (amplicon $\times$ $\log_{10}$ copies/mL)	$2 \times$ stz-MVN$_2(0, 1)$	-0.23 (-0.35, -0.12)	921.03	1843.7	1
α_5 (bait-capture $\times$ $\log_{10}$ copies/mL)	$2 \times$ stz-MVN$_2(0, 1)$	0.23 (0.12, 0.35)	921.03	1843.7	1
σ_{ind}	Half-Cauchy(0,1)	1.57 (1.47, 1.67)	2883.91	4888.79	1
δ_0	Normal(0,3.16^2)	-2.82 (-3.19, -2.45)	4867.72	5010.61	1
β_1 (fishing)	stz-MVN$_3(0, 1)$	0.43 (0.08, 0.79)	5758.71	5376.2	1
β_2 (inland)	stz-MVN$_3(0, 1)$	-0.43 (-0.79, -0.08)	5758.71	5376.2	1
β_3 (sexpever)	Normal(0,1)	0.02 (-0.04, 0.06)	1697.61	3204.27	1
β_4 (fishing $\times$ sexpever)	stz-MVN$_4(0, 1)$	0.02 (-0.03, 0.08)	1699.29	2152.46	1
β_5 (inland $\times$ sexpever)	stz-MVN$_4(0, 1)$	-0.02 (-0.08, 0.03)	1699.29	2152.46	1
logit(λ)	Normal(0,1)[,2.2]	0.51 (0.33, 0.69)	5497.3	5954.7	1
logit(ϵ)	Normal(0,1)	-5.92 (-6.25, -5.58)	4781.63	5538.94	1
