# Supplementary material for: Quantifying prevalence and risk factors of HIV multiple infection in Uganda from population-based deep-sequence data
Source: PLoS Pathog. 2025 Apr 22;21(4):e1013065. doi: 10.1371/journal.ppat.1013065 (PMC12055032; doi:10.1371/journal.ppat.1013065)
Supplement: S15 Table — Excludes participants with ambiguous or missing data on the number of lifetime sex partners. ESS = effective sample size. HPD = highest posterior density. stz-MVN = sum-to-zero multivariate Normal distribution. (PDF) [file ppat.1013065.s028.pdf]

Parameter	Prior	Median (95% HPD)	Bulk ESS	Tail ESS	$\hat{R}$
α_0	Normal(0,2^2)	1.29 (1.14, 1.44)	953.75	1710.24	1
α_1 (amplicon)	$2 \times$ stz-MVN$_1(0,1)$	-1.09 (-1.24, -0.94)	844.8	1972.12	1
α_2 (bait-capture)	$2 \times$ stz-MVN$_1(0,1)$	1.09 (0.94, 1.24)	844.8	1972.12	1
α_3 ($\log_{10}$ copies/mL)	Normal(0,2^2)	1.07 (0.91, 1.23)	913.37	1917.57	1
α_4 (amplicon $\times$ $\log_{10}$ copies/mL)	$2 \times$ stz-MVN$_2(0,1)$	-0.11 (-0.26, 0.04)	1170.16	2045.55	1
α_5 (bait-capture $\times$ $\log_{10}$ copies/mL)	$2 \times$ stz-MVN$_2(0,1)$	0.11 (-0.04, 0.26)	1170.16	2045.55	1
σ_{ind}	Half-Cauchy(0,1)	1.5 (1.38, 1.64)	2808.79	4816.16	1
δ_0	Normal(0,3.16^2)	-2.97 (-3.65, -2.41)	3267.05	3510.48	1
β_1 (fishing)	stz-MVN$_3(0,1)$	0.76 (0.22, 1.4)	3906.19	3940.6	1
β_2 (inland)	stz-MVN$_3(0,1)$	-0.76 (-1.4, -0.22)	3906.19	3940.6	1
β_3 (sexpever)	Normal(0,1)	0 (-0.1, 0.07)	3382.91	2966.97	1
β_4 (fishing $\times$ sexpever)	stz-MVN$_4(0,1)$	0.04 (-0.03, 0.14)	3353.77	2592.48	1
β_5 (inland $\times$ sexpever)	stz-MVN$_4(0,1)$	-0.04 (-0.14, 0.03)	3353.77	2592.48	1
logit(λ)	Normal(0,1)[,2.2]	0.73 (0.41, 1.02)	4232.12	5380.69	1
logit(ϵ)	Normal(0,1)	-5.84 (-6.34, -5.37)	3749.42	4630.76	1
