# Supplementary material for: Quantifying prevalence and risk factors of HIV multiple infection in Uganda from population-based deep-sequence data
Source: PLoS Pathog. 2025 Apr 22;21(4):e1013065. doi: 10.1371/journal.ppat.1013065 (PMC12055032; doi:10.1371/journal.ppat.1013065)
Supplement: S16 Table — ESS = effective sample size. HPD = highest posterior density. stz-MVN = sum-to-zero multivariate Normal distribution. (PDF) [file ppat.1013065.s029.pdf]

Parameter	Prior	Median (95% HPD)	Bulk ESS	Tail ESS	$\hat{R}$
α_0	Normal(0,2^2)	1.23 (1.15, 1.31)	1604.6	2763.04	1
α_1 (amplicon)	$2 \times$ stz-MVN$_1(0, 1)$	-1.21 (-1.29, -1.13)	1583.59	3108.37	1
α_2 (bait-capture)	$2 \times$ stz-MVN$_1(0, 1)$	1.21 (1.13, 1.29)	1583.59	3108.37	1
α_3 ($\log_{10}$ copies/mL)	Normal(0,2^2)	1.2 (1.12, 1.29)	2034.21	3698.21	1
α_4 (amplicon $\times$ $\log_{10}$ copies/mL)	$2 \times$ stz-MVN$_2(0, 1)$	-0.27 (-0.35, -0.19)	1892.01	3456.93	1
α_5 (bait-capture $\times$ $\log_{10}$ copies/mL)	$2 \times$ stz-MVN$_2(0, 1)$	0.27 (0.19, 0.35)	1892.01	3456.93	1
σ_{ind}	Half-Cauchy(0,1)	1.53 (1.47, 1.6)	4403.23	6197.95	1
δ_0	Normal(0, 3.16^2)	-2.92 (-3.29, -2.6)	5292.7	5335.83	1
β_1 (amplicon)	$\tau \times$ stz-MVN$_1(0, \xi_j^2)$	0.01 (-0.11, 0.19)	11235.83	7684.21	1
β_2 (bait-capture)	$\tau \times$ stz-MVN$_1(0, \xi_j^2)$	-0.01 (-0.19, 0.11)	11235.83	7684.21	1
β_3 (2010 survey)	$\tau \times$ stz-MVN$_2(0, \xi_j^2)$	0 (-0.38, 0.33)	9691.25	7054.55	1
β_4 (2012 survey)	$\tau \times$ stz-MVN$_2(0, \xi_j^2)$	0.02 (-0.15, 0.39)	7137.51	6989.4	1
β_5 (2014 survey)	$\tau \times$ stz-MVN$_2(0, \xi_j^2)$	0 (-0.23, 0.31)	9541.22	7195	1
β_6 (2015 survey)	$\tau \times$ stz-MVN$_2(0, \xi_j^2)$	0 (-0.26, 0.2)	7849.31	6749.54	1
β_7 (2017 survey)	$\tau \times$ stz-MVN$_2(0, \xi_j^2)$	0 (-0.27, 0.21)	7891.71	6894.91	1
β_8 (2019 survey)	$\tau \times$ stz-MVN$_2(0, \xi_j^2)$	-0.01 (-0.44, 0.27)	8535.29	5615.45	1
β_9 (uncircumcised men)	$\tau \times$ stz-MVN$_3(0, \xi_j^2)$	0.01 (-0.12, 0.22)	9311.75	7309.43	1
β_{10} (circumcised men)	$\tau \times$ stz-MVN$_3(0, \xi_j^2)$	-0.01 (-0.22, 0.12)	9311.75	7309.43	1
β_{11} (fishing)	$\tau \times$ stz-MVN$_4(0, \xi_j^2)$	0.22 (-0.03, 0.56)	2982.28	5161.98	1
β_{12} (inland)	$\tau \times$ stz-MVN$_4(0, \xi_j^2)$	-0.22 (-0.56, 0.03)	2982.28	5161.98	1
β_{13} (men)	$\tau \times$ stz-MVN$_5(0, \xi_j^2)$	-0.01 (-0.23, 0.11)	9088.21	6648.59	1
β_{14} (women)	$\tau \times$ stz-MVN$_5(0, \xi_j^2)$	0.01 (-0.11, 0.23)	9088.21	6648.59	1
β_{15} ((14,24] years])	$\tau \times$ stz-MVN$_6(0, \xi_j^2)$	0 (-0.23, 0.16)	11041.58	7419.38	1
β_{16} ((24,34] years])	$\tau \times$ stz-MVN$_6(0, \xi_j^2)$	0 (-0.19, 0.17)	10093.38	7413.12	1
β_{17} ((34,49] years)	$\tau \times$ stz-MVN$_6(0, \xi_j^2)$	0.01 (-0.15, 0.22)	11368.69	6877.98	1
β_{18} (non-migrant)	$\tau \times$ stz-MVN$_7(0, \xi_j^2)$	-0.01 (-0.26, 0.11)	8782.71	6380.74	1
β_{19} (in migrant)	$\tau \times$ stz-MVN$_7(0, \xi_j^2)$	0.01 (-0.11, 0.26)	8782.71	6380.74	1
β_{20} (no bar/rest. worker)	$\tau \times$ stz-MVN$_8(0, \xi_j^2)$	0 (-0.14, 0.18)	10611.81	7028.27	1
β_{21} (bar/rest. worker)	$\tau \times$ stz-MVN$_8(0, \xi_j^2)$	0 (-0.18, 0.14)	10611.81	7028.27	1
β_{22} (women $\times$ fishing)	$\tau \times$ stz-MVN$_9(0, \xi_j^2)$	-0.01 (-0.22, 0.13)	10487.4	6942.16	1
β_{23} (men $\times$ fishing)	$\tau \times$ stz-MVN$_9(0, \xi_j^2)$	0.01 (-0.13, 0.22)	10487.4	6942.16	1
β_{24} (women $\times$ inland)	$\tau \times$ stz-MVN$_{10}(0, \xi_j^2)$	0 (-0.2, 0.18)	12788.45	6888.19	1
β_{25} (men $\times$ inland)	$\tau \times$ stz-MVN$_{10}(0, \xi_j^2)$	0 (-0.18, 0.2)	12788.45	6888.19	1
β_{26} ((14,24] years] $\times$ fishing)	$\tau \times$ stz-MVN$_{11}(0, \xi_j^2)$	0 (-0.22, 0.18)	12007.06	7747.6	1
β_{27} ((24,34] years] $\times$ fishing)	$\tau \times$ stz-MVN$_{11}(0, \xi_j^2)$	0 (-0.2, 0.17)	8495.11	7547.93	1
β_{28} ((34,49] years $\times$ fishing)	$\tau \times$ stz-MVN$_{11}(0, \xi_j^2)$	0 (-0.17, 0.23)	11714.28	7616.89	1
β_{29} ((14,24] years] $\times$ inland)	$\tau \times$ stz-MVN$_{12}(0, \xi_j^2)$	0 (-0.28, 0.22)	10500.43	7134.42	1
β_{30} ((24,34] years] $\times$ inland)	$\tau \times$ stz-MVN$_{12}(0, \xi_j^2)$	0 (-0.2, 0.27)	7910.91	6163.02	1
β_{31} ((34,49] years $\times$ inland)	$\tau \times$ stz-MVN$_{12}(0, \xi_j^2)$	0 (-0.24, 0.25)	10568.93	6623.9	1
β_{32} (non-migrant $\times$ fishing)	$\tau \times$ stz-MVN$_{13}(0, \xi_j^2)$	0 (-0.16, 0.19)	11754.24	7247.9	1
β_{33} (in migrant $\times$ fishing)	$\tau \times$ stz-MVN$_{13}(0, \xi_j^2)$	0 (-0.19, 0.16)	11754.24	7247.9	1
β_{34} (non-migrant $\times$ inland)	$\tau \times$ stz-MVN$_{14}(0, \xi_j^2)$	-0.02 (-0.39, 0.12)	7523.96	7241.64	1
β_{35} (in migrant $\times$ inland)	$\tau \times$ stz-MVN$_{14}(0, \xi_j^2)$	0.02 (-0.12, 0.39)	7523.96	7241.64	1
β_{36} (no sex & bar/rest. worker $\times$ fishing)	$\tau \times$ stz-MVN$_{15}(0, \xi_j^2)$	0.01 (-0.11, 0.22)	10596.93	7933.99	1
β_{37} (sex & bar/rest. worker $\times$ fishing)	$\tau \times$ stz-MVN$_{15}(0, \xi_j^2)$	-0.01 (-0.22, 0.11)	10596.93	7933.99	1
β_{38} (no sex & bar/rest. worker $\times$ inland)	$\tau \times$ stz-MVN$_{16}(0, \xi_j^2)$	-0.01 (-0.27, 0.18)	10259.8	6942.55	1
β_{39} (sex & bar/rest. worker $\times$ inland)	$\tau \times$ stz-MVN$_{16}(0, \xi_j^2)$	0.01 (-0.18, 0.27)	10259.8	6942.55	1

τ	Half-Cauchy(0, 1)	0.11 (0, 0.27)	3527.04	3520.19	1
ξ_1	Half-Cauchy(0,1)	0.76 (0, 3.97)	5822.05	4329.7	1
ξ_2	Half-Cauchy(0,1)	0.73 (0, 3.87)	6568.72	5297.73	1
ξ_3	Half-Cauchy(0,1)	0.78 (0, 4.23)	5860.4	4013.51	1
ξ_4	Half-Cauchy(0,1)	0.84 (0, 4.17)	5668.74	4546.22	1
ξ_5	Half-Cauchy(0,1)	0.75 (0, 3.82)	5776.99	4350.27	1
ξ_6	Half-Cauchy(0,1)	0.71 (0, 3.64)	6016.01	3976.24	1
ξ_7	Half-Cauchy(0,1)	0.71 (0, 3.64)	5948.54	4929.5	1
ξ_8	Half-Cauchy(0,1)	0.81 (0, 4.28)	5706.46	4352.22	1
ξ_9	Half-Cauchy(0,1)	0.76 (0, 4.04)	6448.6	5229.87	1
ξ_{10}	Half-Cauchy(0,1)	0.76 (0, 3.84)	5448.99	4109.24	1
ξ_{11}	Half-Cauchy(0,1)	1.54 (0, 6.7)	5204.54	3781.71	1
ξ_{12}	Half-Cauchy(0,1)	1.53 (0, 6.77)	4665.41	3351.82	1
ξ_{13}	Half-Cauchy(0,1)	0.76 (0, 4.02)	5768.59	4750.3	1
ξ_{14}	Half-Cauchy(0,1)	0.77 (0, 3.89)	5912.9	4075.77	1
ξ_{15}	Half-Cauchy(0,1)	0.73 (0, 3.73)	6002.97	4697.86	1
ξ_{16}	Half-Cauchy(0,1)	0.69 (0, 3.9)	5853.28	4854.13	1
ξ_{17}	Half-Cauchy(0,1)	0.72 (0, 3.87)	5761.74	4349.59	1
ξ_{18}	Half-Cauchy(0,1)	0.76 (0, 4.11)	6013.58	4675.29	1
ξ_{19}	Half-Cauchy(0,1)	0.8 (0, 4.1)	5534.17	4086.15	1
ξ_{20}	Half-Cauchy(0,1)	0.71 (0, 3.91)	5822.09	4384.87	1
ξ_{21}	Half-Cauchy(0,1)	0.71 (0, 3.91)	5498.92	4711.74	1
ξ_{22}	Half-Cauchy(0,1)	0.77 (0, 4.14)	6038.43	4710.93	1
ξ_{23}	Half-Cauchy(0,1)	0.75 (0, 4.15)	5848.67	5074.45	1
ξ_{24}	Half-Cauchy(0,1)	0.75 (0, 4.12)	5515.18	4300.96	1
ξ_{25}	Half-Cauchy(0,1)	0.76 (0, 3.96)	5356.71	4352.14	1
ξ_{26}	Half-Cauchy(0,1)	0.74 (0, 3.91)	5643.39	5016.96	1
ξ_{27}	Half-Cauchy(0,1)	0.72 (0, 3.83)	6831.73	5127.73	1
ξ_{28}	Half-Cauchy(0,1)	0.71 (0, 3.81)	5717.59	4881.52	1
ξ_{29}	Half-Cauchy(0,1)	0.78 (0, 3.95)	6008.34	4393.88	1
ξ_{30}	Half-Cauchy(0,1)	0.76 (0, 3.92)	5919.21	4866.68	1
ξ_{31}	Half-Cauchy(0,1)	0.76 (0, 3.92)	5970.57	4938.37	1
ξ_{32}	Half-Cauchy(0,1)	0.76 (0, 4.2)	6007.24	4623.83	1
ξ_{33}	Half-Cauchy(0,1)	0.74 (0, 3.74)	5433	3881.02	1
ξ_{34}	Half-Cauchy(0,1)	0.89 (0, 4.59)	6034.98	4671.29	1
ξ_{35}	Half-Cauchy(0,1)	0.88 (0, 4.55)	5837.69	5137.13	1
ξ_{36}	Half-Cauchy(0,1)	0.78 (0, 3.87)	5827.41	4655.85	1
ξ_{37}	Half-Cauchy(0,1)	0.76 (0, 4.18)	5334.78	4493.09	1
ξ_{38}	Half-Cauchy(0,1)	0.81 (0, 4.34)	5587.07	4553.77	1
ξ_{39}	Half-Cauchy(0,1)	0.82 (0, 4.13)	5844.87	4565.2	1
logit(λ)	Normal(0,1)[,2.2]	0.3 (0.12, 0.48)	4843.14	5191.2	1
logit(ϵ)	Normal(0,1)	-5.73 (-5.98, -5.51)	4808.66	4706.17	1
