# Supplementary material for: Quantifying prevalence and risk factors of HIV multiple infection in Uganda from population-based deep-sequence data
Source: PLoS Pathog. 2025 Apr 22;21(4):e1013065. doi: 10.1371/journal.ppat.1013065 (PMC12055032; doi:10.1371/journal.ppat.1013065)
Supplement: Attachment — Submitted filename: PLOS_Pathogens_response_to_reviewers.pdf [file ppat.1013065.s030.pdf]

We thank the reviewers and the editor for their helpful comments on our submitted manuscript. We feel that the revised manuscript is improved considerably as a result of this feedback. In summary, we made the follow revisions in response to editor and reviewer feedback:

1. We re-framed the text in the Introduction and Discussion to focus specifically on multiple infections that arise due to superinfection and have removed references and text relevant to multiple infections that arise through coinfection at the time of initial infection. We agree with the reviewers that co-infections tend to be genetically very closely related and that our method would not identify these as distinct infections.
2. We have included an additional sensitivity analysis (S10 Table) in which we remove our minor variant frequency-based (1%) filtering threshold. This analysis shows that while the estimated prevalence of multiple infections is slightly higher when this filter is removed, the difference is relatively minimal (5.86% v. 6.1%, Line 502), indicating that this filtering protocol did not significantly bias our estimates downwards through removal of minor variants.
3. We have included an additional sensitivity analysis in which we estimate the prevalence of multiple infections in the study sample using data only from the gp41 and p24 regions used in previous studies of HIV superinfection in this setting (e.g. 10.1093/infdis/jis325). In the absence of amplicon sequence data from the same set of samples, this allows us to compare our estimates of multiple infection based on whole genome data to the estimates that would have been obtained using only amplicon sequencing data. We estimate the prevalence of multiple infections in the study sample based on this synthetic amplicon data to be only 2.31% (Line 508). This highlights the value in including whole-genome sequence data for more sensitive detection of multiple infections.

In addition, we have made the following changes based on internal discussions throughout the review process:

1. To account for any potential biases in our sequenced sample as compared to the underlying population of viremic study participants, we introduce a post-stratification weighting procedure as in 10.1080/01621459.1993.10476368 (eq. 22). This does not significantly affect our previously reported estimate of the prevalence of multiple infections in the study sample (6%). However, because participants living in fishing communities, which have a higher burden of multiple infections, are overrepresented in our sequence data relative to the underlying population (S4 Table), the estimated prevalence in the population is slightly lower (4%, Line 513-514).
2. In light of recently developed features in the Stan programming language (v.2.36 released December 10, 2024) we have adjusted our logistic regression models such that we include a covariate for all levels of categorical variables (Eqs 13 and 14). This ensures that the prior distribution is the same for all levels. We maintain identifiability by constraining the sum of the coefficients to be 0. This change has not significantly affected our primary findings regarding the prevalence of multiple infections in the study sample or risk factors for harboring multiple infections.

Reviewers' Comments:

Part I - Summary

Reviewer #1

Martin and colleagues present a novel method to identify multiple infections in PLWH using whole-genome deep-sequencing data. They use this method to quantify the incidence of multiple infection and infer risk factors associated with it. Their analysis of data sampled from PLWH in Uganda demonstrates a positive effect of high community HIV-1 prevalence on multiple infection acquisition risk. Their Bayesian model is thoughtful to account for partial sequencing and expected false-negative/positive rates, as well as conditioning the prior on viral load and sequencing protocol, and appears to perform well on simulated data. This could be a useful method in light of the proliferation of NGS data for HIV-1. The manuscript is generally well written and the method clearly documented. - Eric Lewitus

We thank the reviewer for their careful reading of our manuscript and the kind words about our work.

Reviewer #2

The Rakai project investigators present here a new study of HIV superinfection. While the findings themselves follow on many similar studies over the years demonstrating superinfection, particularly in high prevalence areas and populations, this study applies newer methods and samples a larger population, making these estimates of superinfection prevalence likely more robust for the population understudy. Overall, the study presents what looks to be technical advance that may be useful for these types of studies going forward although I was not able to evaluate the computational aspects of the method.

It was a bit hard to read as the authors are quite negative regarding earlier work which is a shame given some of it really set the foundation for understanding HIV superinfection.

We thank the reviewer for their effort in reviewing our manuscript. In response to the reviewer comments we have softened the language regarding previous work on understanding HIV superinfection to more accurately reflect the important contributions of that work to our understanding of HIV superinfection, particularly given technological limitations at the time when the work was done. We hope these changes improve the interpretation of our results in light of previous studies.

For example:

Introduction (line 27-29): *"While this approach proved useful prior to the availability 22 of deep-sequencing technologies, it has a sensitivity of only ~ 5% to detect variants present in ≤ 20% of the within-host viral population [24]."*

Introduction (line 32-34): *“This approach is more sensitive in detecting minor variants than bulk sequencing and was considered the “gold standard” [32], but is labor intensive and difficult to apply at scale. Amplicon deep-sequencing of discrete regions of the HIV genome is able to achieve high sensitivity while being highly scalable to large sets of samples and has therefore been broadly applied to study multiple infections in larger studies [3,32,37–39].”*

Part II – Major Issues: Key Experiments Required for Acceptance

Reviewer #1

There is one major source of confusion that makes the manuscript (and its implications) difficult to process. While the authors articulate the distinction between co-infection (when multiple founder variants are established at acquisition) and superinfection (when a secondary infection is acquired, typically months or years following the initial acquisition), they do not clearly address which they are detecting or indeed can detect with their method. They state in the Discussion that they cannot "distinguish multiple infections through coinfection and superinfection", even though differences between these are ostensible and the authors could venture to differentiate between them. If indeed they are aiming to detect both, then their estimate of a 6% rate of multiply infected individuals is very low. However, it seems they are knowingly detecting superinfections (lines 647-651, 750-752) and even, by setting k=0.067 and removing rare variants, excluding co-infections. Perhaps the project started agnostic to the type of multiple infection, but eventually the authors realized they were only detecting superinfections. Fair enough. But for a reader who might try to use this method, it is essential they know what they can detect with it.

Associated risk factors, diversification profiles, effects on nAb development, and rates of occurrence are not always aligned for co-infections and superinfections (some references supplied in itemized comments), so I think it is important the authors clarify this point. Firstly, I suggest the they expand their simulations to identify lower diversity boundaries on the limits of detection with their method and compare those boundaries to typical diversity estimates for co-infections. This can then be applied to their analysis by comparing simulated results to subgraph distances to the MRCA in empirical analysis. Secondly, in light of those simulations, I recommend the authors are more explicit about the types of multiple infections they are detecting and re-center their manuscript on the superinfection literature. A manuscript more attuned to this would be stronger, I think. Alternatively, by setting k above the average distance between multiple founder variants at, say, six months and removing rare variants the authors could simply state that they are intentionally excluding co-infections (or trying to) and focus on superinfections.

While in no way does this caveat undermine the authors' method or results, I think making a harder distinction between co- and super-infections will clarify the applicability of their method and the context of their results.

We again thank the reviewer for their detailed feedback. After careful consideration of the reviewer's comments we agree that given our current model specification (e.g. k=0.067) we are unable to identify co-infection of a given participant with multiple distinct, yet highly related, viral genomes resulting from within-host evolutionary processes within the source partner. Further, we agree that adjusting the framing of our manuscript to focus specifically on superinfections more closely aligns with the presented analysis. To this end, have made the following text changes:

Introduction (line 2-4): *"Simultaneous infection with multiple distinct variants of human immunodeficiency virus (HIV) can occur through a process called superinfection following secondary exposure to infected bodily fluids"*

Discussion (line 709-711): *"Similarly, in the absence of longitudinal data or data sampled soon after initial infection we are unable to reliably distinguish multiple infections acquired through coinfection and superinfection. However, based on our parametrization of the k parameter within phyloscanner and the genetic distance between observed multiple subgraphs, we suspect that the vast majority of identified multiple infections are due to superinfection with a genetically distinct viral genotype. More liberal values of k would increase the sensitivity of our approach to identify closely related viral genotypes (such as those acquired during co-infection) at the expense of an increased rate of false-positives."*

We do note that given the cross-sectional nature of these data our method may identify co-infection of a recipient participant from a source participants who themselves was superinfected (and thus harbors multiple highly divergent HIV genomes), however, this is likely a very rare scenario as compared to "canonical" co-infection with closely related genomes.

In response to the reviewer's specific suggestions regarding our simulation study we note that our simulation framework does not directly simulate viral diversity. Instead, it relies on a pre-defined proportion of genomes that are deemed to be "multiply infected" (M_i in Eq 23a and 24a) agnostic of what that means with regards to genetic divergence) and then simulates the impact of partial sequence success and false positives and false negatives on the expected output data.

However, to address the reviewer's concern we have included an additional supplementary figure (S2 Fig) showing pairwise distance between unique tips in within-host phylogenetic trees colored by whether those tips were assigned to be part of the same subgraph or different subgraphs. This figure shows that phylogenetic tips from different subgraphs are very divergent as compared to those that are identified as part of the same subgraph. Given the small region of overlap between these two distributions, we expect that our results are robust to the exact specification of the k parameter, within a reasonable range. We hope that this additional visualization provides helpful context regarding the genetic differentiation between subgraphs.

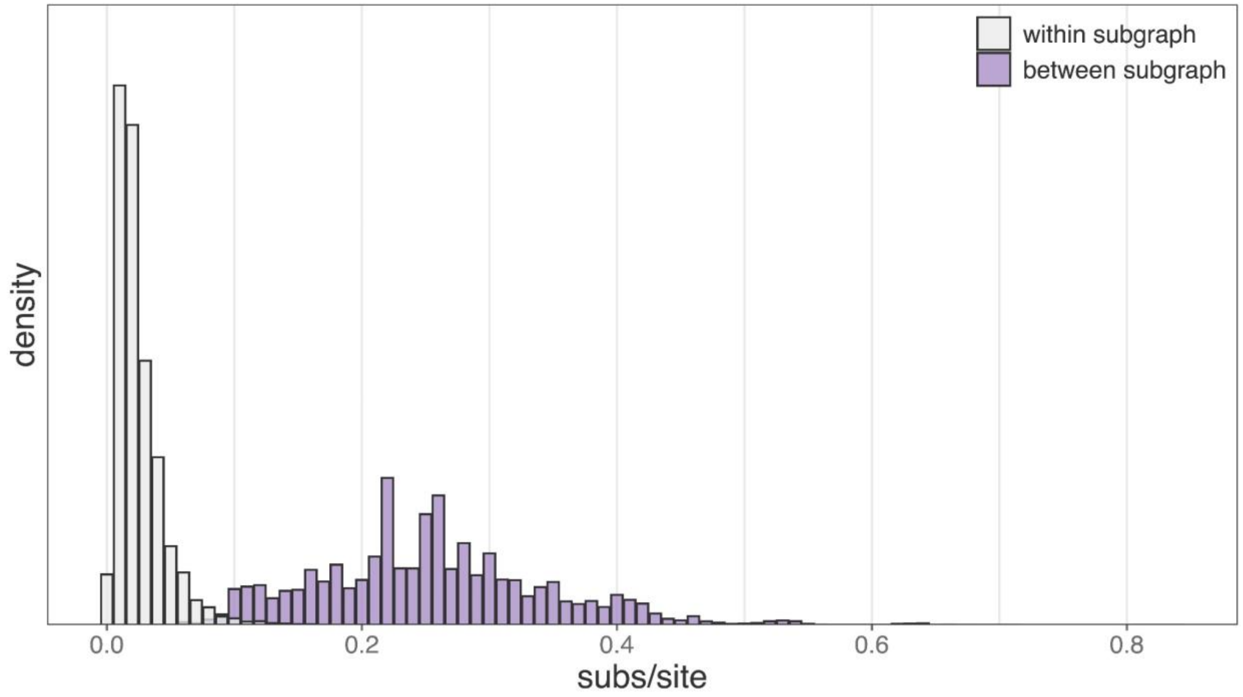

Reviewer #2

No experiments needed specifically although if they do not have data where they can compare their method to other methods, then Mmke clear that this method was not directly shown to be better at detecting superinfection than other methods - that is just an assumption.

We agree with the reviewer that a direct comparison between our methods and other methods would be very informative regarding the potential improvement of the work presented in this manuscript. However, we lack the requisite data, specifically amplicon sequence data as has been used in previous studies of HIV superinfection.

However, to address the reviewer's concerns to the extent that it is possible with existing data we simulated synthetic amplicon data from our deep-sequence data for the same genome regions as included in Redd 2012 (10.1093/infdis/jis325, S5 File). Of those samples with available sequence data in either *p24* or *gp41*, 4.31% had multiple subgraphs in one of the regions as compared to 8.92% based on whole-genome data. When fitting our model to the synthetic amplicon data we estimate the prevalence of multiple infections in the study sample to be only 2.3% as compared to 6% when estimated using whole-genome data. We therefore conclude that inclusion of whole-genome data is more sensitive in identifying multiple infections.

Results (line 439-440)*: "Of 1,742 samples with data in windows spanning the p24 (1427 - 1816) and gp41 (7941 - 8264) regions 75 (4.31%) had multiple subgraphs in one of the regions."*

Results (line 502-509): *"When considering only genome windows spanning the p24 and gp41 regions as in previous studies (e.g. [3,37,38]), we were unable to estimate σind with suitably*

high effective sample size (ESS) values as there were at most two regions of data for each sample. We therefore fixed σind = 0.7 based on the whole-genome analysis (S9 Table) and found that that the sample prevalence of multiple infections based on p24 and gp41 was considerably lower as compared to the whole-genome analysis (2.31% [0.71% - 4.94%], S10 S11 Table), highlighting the utility of incorporating whole-genome data into our inference.“

Note the limitations of cross sectional studies for understanding correlates

We have referenced limitations of cross-sectional studies for understanding risk factors in the Discussion.

Discussion (line 707-709): *“Further, with only a single sample per-individual we are unable to reliably identify factors causally associated with incident multiple infection [126] and therefore report factors that are associated with prevalent multiple infections.”*

Part III – Minor Issues: Editorial and Data Presentation Modifications

Reviewer #1

Additional comments are listed as they appear in the manuscript.

Line 6: references 3 & 4 refer to superinfection only

Given the reframing of the manuscript to focus on superinfections this comment is no longer relevant.

Line 11: co-infection and superinfection have also been shown to augment nAb development (e.g., for co-infection: Lewitus et al, PLoS Path, 2022; for superinfection: Powell et al., J Vir, 2010; Cortez et al., J Vir, 2011; Krebs et al., Immunity, 2019), although this is less consistent for superinfections.

We thank the reviewer for highlighting this implication of co-infections and superinfections. We have added additional references on the impact of superinfections on nAB development.

Introduction (line 11-14): *“Acquisition of superinfections may also increase the breadth and strength of the antibody response to HIV infection [14 - 16], potentially aiding in the identification of broadly neutralizing antibodies [17].”*

Lines 47-49: would help to mention that Piantadosi et al's inability to identify superinfection in env was due to recombination (not because infecting envs were similar)

Based on the results presented results in Piantadosi 2008 and Chohan 2005 we do not believe it is clear whether the lack of genetic diversity in *env* at the two time points presented in Chohan

2005 for the individuals discussed in Piantadosi was due to within-host recombination or superinfection with a circulating recombinant virus. To make this distinction, it would be necessary to show that at intermediate time-points there was genetic diversity present in *env* that was eliminated through within-host recombination and sweep of that recombinant virus (such that diversity remained in *gag* as shown in Piantadosi but not in *env* as shown in Chohan). To our knowledge this analysis has not been presented and it is only evident that the two people discussed in Piantadosi were superinfected at some point with a virus that was distinct from their initial infecting strain in the *gag* region. We have therefore left the text as is.

Line 58: please mention some of the methods that exist for identifying co-infections (e.g., Keele et al., PNAS, 2008; Dearlove et al., PLoS CompBio, 2021; Lewitus & Rolland, Virus Evolution, 2019).

Given the reframing of the manuscript to focus on superinfections this comment is no longer relevant.

Line 61: What does "phylogenetically likely" mean? Likely based on the phylogeny?

We agree with the reviewer that the use of this phrase is confusing to the reader. We have revised this sentence for clarity.

Results (line 515-516): *"We next used our model to identify individuals with likely multiple infection based on their within-host phylogenetic trees and our modeling framework"*

Line 67: introduce RCCS acronym

We thank the reviewer for catching our missing introduction of the RCCS acronym and have adjusted this in the text.

Lines 84-87: this mostly repeats the top of the paragraph

We have shortened this sentence for brevity and to avoid repeating content from earlier in the paragraph.

Introduction (line 79-81): *"We validated model performance on simulated data and used it to identify multiple infections in RCCS participants over a period of declining incidence and rapidly shifting transmission dynamics [43,46]"*

Line 198: Was 250 bp chosen because it was the minimum read length? If so, please state that.

What is the expected effect of using sequencing protocols with longer read lengths (e.g., Nanopore or PacBio)?

Details on the optimal choice for the window size in phyloscanner analysis can be found in 10.1093/molbev/msx304. In short, the optimal read length is long enough to capture within-host diversity but short enough such that some reads fully span the window and that recombination is not expected to have obscured the phylogenetic signal. In our analysis we have found 250 bp to be the optimal choice for our short-read HIV deep-sequence data.

Longer read lengths would improve the phylogenetic resolution within a given window which would, in theory, reduce the false negative rate in our inference of multiple subgraphs. However, we have not generated long-read data for this cohort and are therefore unable to assess this using real-world data. A detailed analysis on the impact of long-read data on the ability to infer transmission directionality using phyloscanner can be found in 10.1101/2023.10.19.563197.

Methods (line 175-178): *"As suggested in [45], this window-size was chosen to be long enough to capture sufficient within-host diversity to provide phylogenetic signal but no longer than the target read length and short enough to minimize within-window recombination."*

Lines 199-201: Were just HVs removed? Based on Fig 1, it looks like most of gp120 is absent.

We have adjusted the text to more accurately describe which windows were excluded from the analysis.

Methods (line 178-180): *"Windows spanning env gp120 were excluded as genetic diversity in the variable loop regions [52] led to poor sequence alignment and unreliable within-host phylogenetic trees."*

Lines 203-4: How were the RCCS sequences subtyped?

In this work we do not present any results based on the subtyping of RCCS sequences. Representative circulating subtypes in the included reference sequences were based on previous analysis of circulating subtypes in Rakai and generally throughout sub-Saharan Africa. A detailed analysis of subtypes circulating in Rakai can be found in 10.1093/ve/veae065.

Lines 216-244: It would be helpful to have a schematic, at least as a supplement, for the protocol described here.

In order to avoid duplicating content across manuscripts we point the reviewer to the original Phyloscanner publication (10.1093/molbev/msx304, in particular Supplementary Information 1.2 and Supplementary Figure 1) as the analysis describe within these lines was conducted entirely within Phyloscanner and is not a novel procedure for this manuscript. This has been clarified in the text in this section of the manuscript.

Methods (line 201-204): *"To accurately assign nodes without relying on patterns of phylogenetic clustering with reference sequences, we employed a modified Sankoff minimum parsimony*

algorithm for ancestral state reconstruction as described in [38,57] (in particular, see Supplementary Information 1.2 and Supplementary Figure 1 in [38]).”

Lines 238-240: I was confused by how k was parameterized. Accounting for divergence between variants prior to the host becoming infected assumes a co-infection, right? Or is this divergence from a reference? Or divergence from a transmission pair? Some clarifying language would be helpful. I think I'm getting stuck on what divergence is being referred to "prior to a given host becoming infected". Also, please try to use acquisition rather than infected when appropriate.

We agree with the review that the methods described here are complicated and potentially confusing. We have adjusted the text for clarity. The reviewer is correct that accounting for divergence that occurred prior to the host becoming infected would assume a co-infection.

Methods (line 212-223): *“Traditional parsimony is recovered when $k = 0$ which will always assign all tips in a single-host tree to a single subgraph, regardless of the phylogenetic branch length captured within that subgraph. As $k \to \infty$, each tip belonging to host h will be assigned to a unique subgraph. Here, we parameterized k with the goal of distinguishing evolution that occurred within a given host from evolution that occurred prior to HIV acquisition, in the case of multiple infection. In the case of single infection, all tips in a single-host tree will be closely related (e.g. Fig 1A) and therefore we want the ancestral reconstruction that minimizes c(n, h) to assign all tips to a single subgraph. In the case of a multiple infection the tips will be expected to fall into (≥)2 clades with relatively small within-subgraph distances but large between-subgraph distances and we seek to parameterize k such that the ancestral reconstruction minimizing c(n, h) differentiates these clades into distinct subgraphs.”*

Line 241: I think k=0.067 would exclude most co-infections, certainly if sampled during acute infection.

We agree with the reviewer that our parameterization k would likely exclude most co-infections with multiple transmitted/founder variants at the time of initial infection. In response to the reviewer’s comments we have reframed our introduction to focus specifically on superinfections.

Lines 246-249: Rare variants can be common in co-infections (Yifan Li, Dynamics Conference, 2024). This step therefore may be discarding co-infections. I think that is fine, but the authors should be clear that they are deliberately excluding possible co-infections.

We expect that co-infections would be sufficiently closely related to each other such that they would not be split into a separate subgraph using our current parameterization of k. To quantify the sensitivity of our results to our filtering protocol, we re-estimated the prevalence of multiple

infection without our frequency-based filtering (while keeping the minimum read count threshold to avoid overestimating multiple infections due to sequencing error). We find that the estimated prevalence in the study samples increases only slightly in the absence of the frequency-based filtering (from 5.88% to 6.10%) indicating that the removal of any potential rare variants in our sequencing data was not biasing our results. However, we cannot account for the presence of rare variants which may not be identified in the sequencing reads.

Results (line 500-502): *"Relaxing our minor subgraph frequency-based filtering step resulted in only a slightly higher prevalence of multiple infections in the study sample (6.1% [4.86% - 7.39%], S10 Table)."*

Line 435: How many sequences per individual?

When multiple samples per individual were available we included only one sample per individual in our analysis of multiple infections as described in S1 File.

Line 455-6: 11% and 19% of 29 windows is 3 and 5.5, respectively. Where do the 2 and 3 come from?

We agree with the reviewer that as written this is confusing. While there are 29 genome windows due to partial sequencing success data is only available for a subset of these windows for some samples (see Figure 1D x-axis). The 11% and 19% statistics in this line refer to the proportion of windows with sequence data for each participant that have evidence of multiple subgraphs, not the proportion of all windows that have evidence of multiple subgraphs. In other words, the denominator for each participant in calculating the percentage is different. We have adjusted the text on line 456 for clarity.

Results (line X): *"Among these, the proportion of sequenced windows in which multiple subgraphs were observed varied considerably, but was generally relatively rare (median [IQR] 11.11 [19.14]% of sequenced windows for each sample, 2 [3] windows total)."*

Line 462: How many individuals were missing gag, env, and nef?

These data are visualized in Fig 1F where the maximum number of sequenced samples within a given window is 2,029. In short, sequencing success was higher in *gag* (79-92% of all samples, dependending on the window) as compared to *env* (48-64%) and *nef* (50%).

Lines 467-469: This is an important result, but points to the confusion in not distinguishing between co-infections and superinfections. The observed rate of coinfection is 25% (see Baxter et al, Lancet Microbe, 2023). If accounting for partial sequencing success serves to more identify multiple infections missed by other methods (or bulk sequencing), then it doesn't make sense that the derived rate here is so low if the authors are considering co- and super-

infections. Which is why I think it's critical they clarify what they're detecting.

Given the reframing of the manuscript to focus on superinfections this comment is no longer relevant.

Fig 1C: Is this averaged across gene windows?

This figure includes data from all observed multiple subgraphs across all genome windows. However, we note that genetic distances in our inferred phylogenetic trees are adjusted to account for differential rates of evolution across the genome (S3 File).

Fig 1F: See my previous comment about gp120 being removed or just HVs?

We have responded to this comment above.

Lines 483-485: If the authors are claiming to detect co-infecions as well, then I suggest they compare their method to existing ones (e.g., Keele et al., Gap Procedure, MGL).

Given the reframing of the manuscript to focus on superinfections this comment is no longer relevant. Further, we have included a comparison to putative results that would have been obtained were sequence data only available from two commonly-used amplicon regions of the HIV genome (see above).

Fig 2A: why the switch from purple to red in depicting multiply infected?

Throughout we use the red color to represent true multiple infections (e.g. in our simulated data). As this is an unobserved variable for the empirical data we use purple to represent instances where there is evidence of multiple subgraphs in the phylogenetic trees for a given sample or within a given sample. Because these multiple subgraphs may be due to a false positive or because multiply infected samples may have no evidence of multiple subgraphs due to partial sequence success or false negatives, we feel that using a distinct color to represent this state is more straightforward to the reader. We have clarified this distinction in the legend to Figure 2.

Fig 2C-H: How should we interpret the x axes?

These figures and similar figures throughout should be interpreted as histograms in which the x-axis is the parameter being plotted and the y-axis is the posterior probability of that parameter

value. The shading is used as a visual indicator of where the 50% and 95% highest posterior density resides. E.g. in Fig 2H the x-axis is the estimated prevalence of multiple infections.

Fig 2E,F: panel labels are incorrect in legend

We thank the reviewer for their meticulous reading of the manuscript. We have corrected the incorrect figure legends.

Line 526: The authors could contextualize this in the Discussion in other estimates of superinfection (I think 3-8% is typically cited, but as the authors have shown this will no doubt vary by community)

We agree with the authors that it is important to contextualize novel results with existing literature. In the revised manuscript we highlight that our estimated prevalence of multiple infections in inland communities is less than in previous studies in this setting, consistent with falling incidence over time due to scale-up of treatment and prevention measures. Additional contextualization, particularly to other geographic settings such as the United States, is presented in the Discussion, see lines 507 - 527.

Discussion (line 633-635): *"Multiple infection among inland community study participants in this study (3.14%) was slightly less prevalent than in this earlier work (pre-2009, 4.7% [2]), consistent with reductions in HIV incidence over the same time frame [35]."*

Fig 3A: I don't think this is a clear presentation of these data. Perhaps the authors could ln-transform the posterior probabilities to show any small shifts in median values?

We have chosen to retain plotting these on the natural scale in the main figure, as this scale more clearly differentiates between samples with a high probability of multiple infections v. those with a very low estimated probability of being multiply infected in comparison to other scales. However, we agree with the reviewer that this visualization fails to demonstrate small differences in the posterior probabilities. We have therefore included a figure on the log scale as S6 Fig.

Line 602: Fig 4C reports logistic coefficient - how does this relate to RR median?

As this is a multivariate logistic model the RR associated with each category will depend on the combination of covariates in a given reference and comparator group. As it does not make sense to compare all the RR for all various combinations of parameter values to the same reference group (e.g. we do not feel that comparing Fishing community men to Inland community women is an informative comparison) and we do not wish to use multiple reference groups in a single figure we instead choose to plot the logistic coefficients associated with each

parameter which do not depend on the level of the other parameters in the model. We report the multivariate risk ratio comparing individuals in fishing to inland communities in the text:

Results (line 585-590): *"This analysis confirmed residency in fishing communities as a risk factor of multiple infection among sequenced participants, albeit with a wide credible interval, (multivariate RR median [95% HPD] 1.59 [0.92 - 2.85]), but did not identify any other variables that were associated with significantly higher or lower risk of multiple infection in our sample (Fig 4C and S16 Table)."*

Lines 640-1: Do any gene windows (or combination of windows) perform as well as WGs?

As 180 samples have multiple subgraphs in at least one of the 29 non-overlapping windows (line 452) but the number of samples with multiple subgraphs in any given window (Fig 1G) is between 10 and 30 in most windows we suspect that using data from only one or a small number of windows would have considerably lower sensitivity to identify multiple infections than our whole-genome data. For example, in the genome window with the highest proportion of multiply infected samples, 800 to 1049, only 3.38% (63 / 1862) of samples with available sequence data have multiple subgraphs, before correcting for potential false positives. This is considerably lower than the estimated prevalence of multiple infections using the whole-genome sequence data (6%).

Reviewer #2

The discussion mentions differences in prevalences of superinfection in this versus other studies but they dont discuss some important aspects- namely that people now are often treated and HIV spread in general is lower - both first and second infections as a result. In many African cohort studies, HIV incidence has been declining steadily since ART. Can they estimate exposure in this group versus the earlier groups studied in any way to address this?

We thank the reviewer for highlighting this important epidemiological context. To address the fact that the prevalence of viremic HIV in this setting has fallen considerably over time (2023.10.14.23297021), we include a feature for the survey round in which samples were collected in our multivariate risk factor model (Fig 4C). We do not observe a difference in the prevalence of multiple infections across survey rounds which occurred over a period of approximately 7 years. However, we note that the data included in the study referenced in line 634 (reference [2]) was collected during an even earlier time frame (pre-2009). We have highlighted this in the revised text.

Discussion (line 633-635): *"Multiple infection among inland community study participants in this study (3.14%) was slightly less prevalent than in this earlier work (pre-2009, 4.7% [2]), consistent with reductions in HIV incidence over the same time frame [35]."*

Related to the point above, it is important to make clear the superinfection estimate is for this cohort in this time period. It is not applicable to other cohorts.

We wholeheartedly agree with the reviewer that our prevalence estimates are specific to this setting over the time period in which samples were collected. We have further emphasized this in the Abstract, Introduction, and Discussion.

Abstract: *"We estimated that between 2010 and 2020, 4.09% (95% highest posterior density interval (HPD) 2.95% - 5.45%) of participants in this study with viremic HIV had a multiple infection at time of sampling."*

Introduction (line 58-64): *"Here, we identify individuals that are likely to have multiple HIV multiple infection at the time of sampling, provide minimum estimates of the prevalence of HIV multiple infections in Rakai, Uganda between January 2010 and November 2020, and characterize risk factors for harboring a multiple infection based on HIV RNA deep-sequence data obtained from plasma samples of 2,029 people living with viremic HIV aged 15-49 who participated in the longitudinal, population-based Rakai Community Cohort Study (RCCS) [35, 36]."*

Discussion (line 608-611): *"Among viremic participants in this study over the study period, the estimated prevalence of multiple infections was approximately 4%, reflecting the prevalence of co-circulating multiple infections present at time of sampling."*

The authors make the point this method improves detection but although this seems plausible, that is just a hypothesis at this point. The true test of this would be to apply this method to samples from a previous study and this should be noted.

We agree with the reviewer that our claim that our method improved detection in our initial submission was not directly supported by the analysis we presented. Unfortunately, we lack whole-genome sequence data for the samples included in previous analyses of superinfection in this cohort and lack amplicon sequence data for the samples included in the current study. Consequently, a direct comparison is not possible.

However, to address the concern we have generated synthetic amplicon sequence data from our whole-genome data covering the same amplicon regions as previous studies of superinfection in this setting. Based on this analysis we estimate the prevalence of multiple infection in the study sample to be only 2.3%, considerably lower than the estimate of 5.88% when using whole-genome data.

Results (line 502-509): *"When considering only genome windows spanning the p24 and gp41 regions as in previous studies (e.g. [3,37,38]), we were unable to estimate σind with suitably high effective sample size (ESS) values as there were at most two regions of data for each sample. We therefore fixed σind = 0.7 based on the whole-genome analysis (S9 Table) and found that that the sample prevalence of multiple infections based on p24 and gp41 was considerably lower as compared to the whole-genome analysis (2.31% [0.71% - 4.94%], S10 S11 Table), highlighting the utility of incorporating whole-genome data into our inference."*

The authors should also discuss the limitations of a cross sectional study

We have added additional commentary on the limitations of a cross sectional study to the Discussion.

Line 707-709: “Further, with only a single sample per-individual we are unable to reliably identify factors causally 570 associated with incident multiple infection [121] and therefore report factors that are associated with prevalent multiple infections. “

Note: I can’t evaluate the model aspects of this so have no comments there. But this aspect needs to be evaluated by someone who can.

We thank the reviewer for their helpful comments which have improved the manuscript.
